# Structural and Electronic Properties of Novel Azothiophene Dyes: A Multilevel Study Incorporating Explicit Solvation Effects

**DOI:** 10.3390/molecules29174053

**Published:** 2024-08-27

**Authors:** Laura Vautrin, Alexandrine Lambert, Faouzi Mahdhaoui, Riad El Abed, Taoufik Boubaker, Francesca Ingrosso

**Affiliations:** 1 Université de Lorraine and CNRS, Laboratoire de Physique et Chimie Théoriques UMR 7019, F-54000 Nancy, France; 2 Laboratoire de Chimie Hétérocyclique, Produits Naturels et Réactivité (LR11SE39), Faculté des Sciences de Monastir, Université de Monastir, Avenue de l’Environnement, Monastir 5019, Tunisia

**Keywords:** azothiophene dyes, UV/VIS spectra, solvation, quantum chemistry, QM/MM, molecular dynamics

## Abstract

Among azobenzene derivatives, azothiophenes represent a relatively recent family of compounds that exhibit similar characteristics as dyes and photoreactive systems. Their technological applications are extensive thanks to the additional design flexibility conferred by the heteroaromatic ring. In this study, we present a comprehensive investigation of the structural and electronic properties of novel dyes derived from 3-thiophenamine, utilizing a multilevel approach. We thoroughly examined the potential energy surfaces of the E and Z isomers for three molecules, each bearing different substituents on the phenyl ring at the para position relative to the diazo group. This exploration was conducted through quantum chemistry calculations at various levels of theory, employing a continuum solvent model. Subsequently, we incorporated an explicit solvent (a dimethyl sulfoxide–water mixture) to simulate the most stable isomers using classical molecular dynamics, delivering a clear picture of the local solvation structure and intermolecular interactions. Finally, a hybrid quantum mechanics/molecular mechanics (QM/MM) approach was employed to accurately describe the evolution of the solutes’ properties within their environment, accounting for finite temperature effects.

## 1. Introduction

Azobenzene derivatives possess versatile properties that are extensively exploited in technological applications, ranging from material and polymer chemistry to photomechanics, biotechnology, and biomedicine [1]. Such technologies rely on the Z/E photoisomerization process occurring upon irradiation with visible (VIS) or ultraviolet (UV) light, originally discovered in 1937 for azobenzene [2], and the most recent advances have pushed toward the synthesis of compounds that can undergo such a process by irradiating in a lower energy region of the spectrum [1], paving the way to more extensive biological applications.

Notwithstanding the long history of photochemical applications, such molecules have been known since the end of the XIX century as dyes [3]. The first reports of azo dyes concern compounds that were named Aniline Yellow and Bismarck Brown, obtained in the quest for stable, easily synthesized molecules that could replace natural pigments in industrial processes. At the same time, another important discovery that made the design of azo dyes even more versatile was that of diazonium salts [4].

A relatively younger class of dyes, introduced into commerce in the early 1980s, are thiophene-based azo dyes, conceived as bathochromic azo dyes to obtain the colors blue, green, navy, and black [5]. They were chosen due to their high degree of brightness and the less expensive preparation procedure compared with antrachinone dyes, and the smaller structure of the heteroaromatic ring provides better dyeability and fast sublimation [6]. The absorption band appearing in the electronic spectra in the visible region has been assigned to π to π* transitions involving the whole conjugate system (the two aromatic rings and the diazo group [6]. In photochemistry applications, it is possible to modulate the energy required for photoswitching by selecting electron-rich heterocycles or introducing π-electron-donating substituents at the phenyl ring [7]. Moreover, it has been pointed out that electron-withdrawing groups in the ortho and para positions allow better bathochromic effects [8].

**Figure 1:**
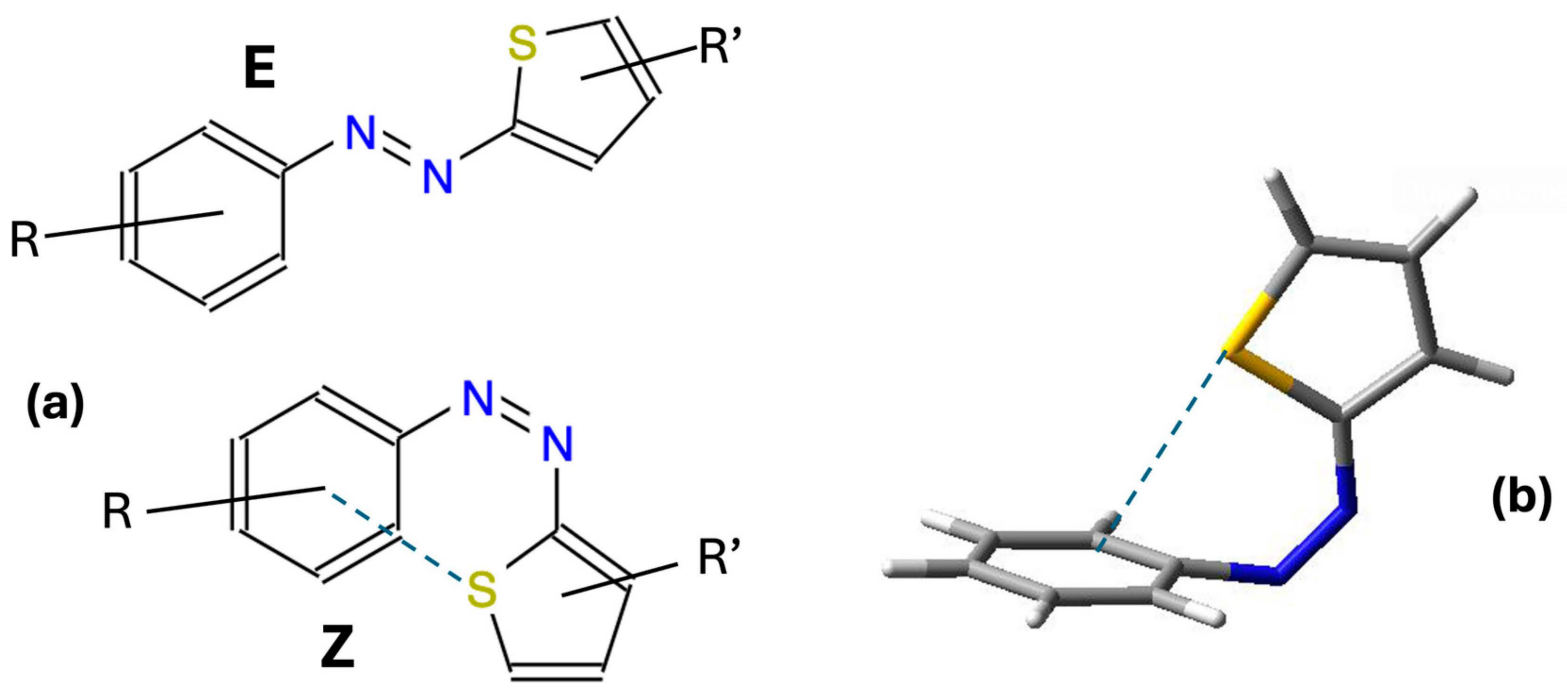
(**a**) Structures of the E and Z isomers of azothiophenes, created using the JSME structure editor [14]. (**b**) Intramolecular interaction (represented using dashed lines) between the lone pair of the S atom on the heteroaromatic ring and the π electron cloud of the phenyl ring, stabilizing the Z isomer.

Despite the interesting properties imparted by the presence of the heteroaromatic ring, azothiophene dyes are relatively less studied than the more popular azobenzene derivatives. In the past few years, there has been an increasing interest in these compounds, bringing important insights into their characteristic properties [7,9,10]. As a matter of fact, in addition to well-separated absorption bands for the Z and E isomers and a stabilized Z isomer, they may be designed to respond to the requirement of lower energy for the photoswitch process in in vivo applications. In azobenzene derivatives, it has been shown that an improvement of the photoswitching process can be achieved, for instance, by activating a radical isomerization pathway and using red light (see [11,12] and references therein). In N- and S-heterocyclic dyes, lone pair-π and -CH-π interactions lead to the thermal stabilization of T-shaped Z isomers (see Figure 1), which therefore display longer lifetimes [4,9,13].

Azothiophene dyes can be prepared by means of a coupling reaction of substituted diazonium salts, already successfully employed in kinetic studies focusing on the nucleophilic nature of 3-substituted thiophenes [15], and similarly to the procedure devised for N,N-disubstituted 2-aminothiophenes [16]. In the present work, we target new diazocompounds synthesized from 3-aminothiophene (IUPAC name: 3-thiophenamine), with particular attention to the structure, the relative energies of the possible isomers, and the absorption properties. A kinetic study of the reactivity leading to the azothiophene compounds was also carried out by some of us and will be published in a separate study, which is in preparation.

We aim to provide further insights into the structure-to-property relation in these compounds and the environmental effects on such properties. In particular, we develop a multilevel strategy to assess the impact of the protonation state of the amino group and the nature of the substituent in the para position on the phenyl ring (i) on the local solvation structure (ii) on the molecular structure along the potential energy surface (PES) (iii) on the π→π* band of the electronic spectra and the frontier orbitals. Our calculations were tailored to the experimental conditions (1 bar, 298 K, solvation medium: a mixture of water and dimethyl sulfoxide (DMSO)) and comprised electrostatic calculations in a continuum, molecular dynamics (MD) simulations of the dyes in the mixture, and subsequent hybrid quantum mechanics–molecular mechanics (QM/MM) MD simulations. To our knowledge, such a detailed picture of the environmental effects on the structural and electronic properties of heteroaryl diazo compounds based on thiophene has not been proposed in the literature yet.

This paper is organized as follows. In Section 3, we describe the computational strategy and the experimental conditions used to measure the UV/VIS spectra. Results are discussed in Section 2. Finally, in Section 4, we provide a summary of our work and some perspectives about ongoing research.

## 2. Results and Discussion

We start our discussion by presenting the results obtained for the minima of the potential energy surface of the three molecules considered in the deprotonated state at the B3LYP/6-311G(d,p) level. All the details and the acronyms concerning the methods can be found in Section 3. The computed energies and enthalpies are reported in Table 1 with respect to the corresponding values obtained for the E isomers.

The picture that we find is consistent with that described in ref. [9] for azothio compounds with the same structure on the phenyl side and a different structure on the thiophene side. In that case, the thiophene ring did not present a substituent, like the amino group in our case. The E isomer is the most stable structure, and another minimum is found, still displaying an E arrangement with respect to the N-N bond but with a different orientation of the thiophene ring (E-twist, Figure 11, top panel). In the case of the Z isomers, a Z-twist arrangement can also be observed as a minimum, but this structure is much more energetic compared with Z (Figure 11, bottom line). Interestingly, we find a difference in the relative energies of these two structures compared with those treated in ref. [9]. In the absence of the amino group, the Z-twist structure is much less destabilized and closer in energy to Z. We can attribute such differences to the steric hindrance of the amino group, leading to a more distorted Z-twist structure. On the other hand, the stabilizing role of the interaction between the S atom and the phenyl ring, which has been characterized as a lone-pair–π interaction existing in other azo heteroaryl compounds [9,13], is confirmed in our work for the E-twist structure but it is less pronounced for Azothio-a. The destabilization of the Z isomer with respect to E is even larger for the molecules bearing substituents in the para positions (Azothio-b and -c). In all cases, the presence of the amino group gives rise to additional π interactions on the thiophene ring, thus making the lone pair of the S atom less available. To provide a complete picture of the PES, we performed a transition state search for the isomerization process. The values of the barriers obtained thanks to these calculations are reported in Table 2.

**Figure 2:**
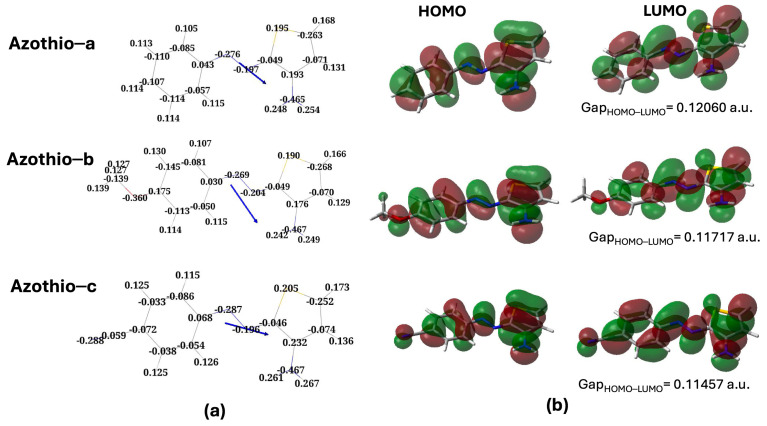
(**a**) Mulliken charges (in a.u.) and direction of the molecular dipole vector (not in scale) for the three E isomers. The dipole moment values are 5.6 D, 2.7 D, and 12.6 D for Azothio-a, Azothio-b, and Azothio-c, respectively. (**b**) Visualization of the frontier orbitals of the three molecules and computed HOMO-LUMO gaps. Results from B3LYP/6-311G(d,p) calculations.

As expected, at ambient temperature and pressure, the E isomer is largely predominant. From now on, we focus on the analysis of the electronic properties of this structure for the three molecules considered. In Figure 2a, we report the Mulliken atomic charges and the direction of the molecular dipoles. The dipole moments are 5.6 D, 2.7 D, and 12.6 D for Azothio-a, -b, and -c, respectively.

The presence of the electron-donating group -OCH_3_ leads to a lower dipole moment compared with Azothio-a, whereas the opposite effect is observed in the presence of the electron-withdrawing group -CN. The strong polarization along the C-N axis induces a change in the direction of the molecular dipole moment and larger changes in the partial changes in key atoms (the two N from the azo group and the S atom of thiophene). The partial charges distribution on the amino group, on the other hand, results are very similar in all cases, the only exception being a slightly more acidic character of the H amino atoms in Azothio-c.

Turning to Figure 2b, we analyze the key features of the frontier orbitals of the E isomers. The abbreviations used are the conventional acronyms, HOMO, for the Highest Occupied Molecular Orbital, and LUMO, for the Lowest Unoccupied Molecular Orbital. In all cases, we note π orbitals which are remarkably similar to those reported for pyrrole-based azoheteroarenes [13]. However, we can clearly see the contribution of the N atom in the amino group to the π system of the two orbitals, leading to a local antibonding character in both cases. The overall shape of the orbitals is the same independently of the substituent but, as expected, we note remarkable changes around the substituents themselves. In Azothio-b, the O atom participates in the π system with a 2p atomic orbital (lone pair), with an antibonding contribution (HOMO, LUMO). This is similar to what was observed in Azothio-c, this time for a bonding π orbital of the C-N bond. In the latter case, the deformation of the local density on the phenyl ring is larger than in the two other cases.

**Figure 3:**
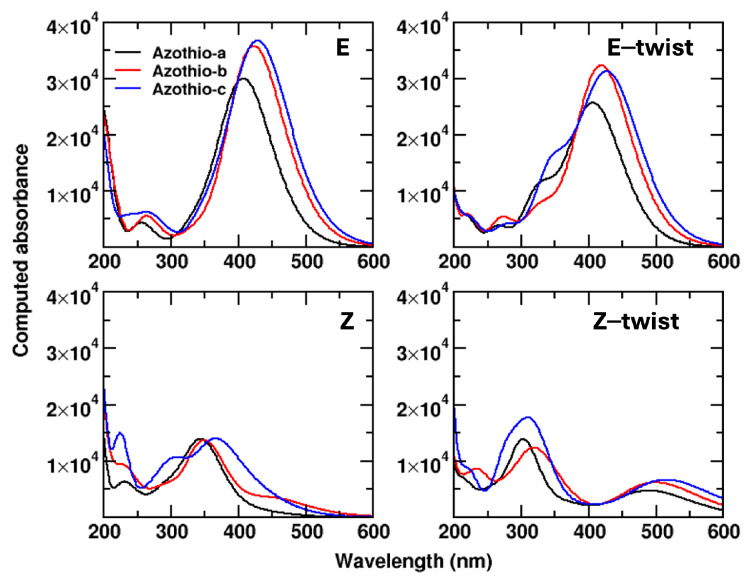
Absorption spectra of neutral species at the B3LYP/6-311G(d,p) level.

Having this picture in mind, we move to comment on the results that we obtained for the absorption spectra, reported in Figure 3.

The first remark is that, not surprisingly, the position of the maximum in the absorption band ($\lambda_{MAX}$) correlates with the HOMO-LUMO gap (values reported in Figure 2): the higher the value of $\lambda_{MAX}$, the lower the gap. The lower value of $\lambda_{MAX}$ is observed for Azothio-a (407 nm), then we have Azothio-b (422 nm), and finally, Azothio-c (430 nm). This band is related to HOMO-LUMO transitions, thus involving the π system. Thanks to the results reported as Supplementary Materials to ref. [9], we were able to make a qualitative comparison with experiments for the spectra for the E and the Z isomers, though we have to remember that the thiophene ring did not bear any substituent in that study. In the experimental results, moving from the system with no substituent on the phenyl group to the other two, a bathochromic shift is observed (about 30 nm, in agreement with what we found).

Some further insights are brought by the comparison of the spectra for the Z isomer. The experimental results [9] point to a hypsochromic shift for the three molecules, to a very similar value of $\lambda_{MAX}$, and to a lower intensity of the band. For our molecules, we have a larger hypsochromic shift (about 60 nm), with the exception of Azothio-c (44 nm), and we also see a decreased intensity. The spectra for Azothio-a and b- are very similar. Keeping in mind that the experimental systems are slightly different, the qualitative agreement that we obtain is satisfying. Some relevant differences are observed between the Z and the Z-twist spectra, which are basically related to the first two transitions. These π→π* transitions are more complex than in the case of the E isomers, since they involve more molecular orbitals (from HOMO−2 to LUMO+1). In the case of HOMO, HOMO−1, and LUMO, the contribution of the amino group is important, thus leading to different transition energies in the Z isomer (with the amino group pointing away from the π system of the aromatic ring) compared with Z-twist (with the amino group interacting with such a π system).

To conclude, we note that the shape of the band is slightly different in the case of the E-twist structure, since the higher energy portion of the band contains a shoulder, not seen in the E case. Such shoulder is related with a HOMO−2 to LUMO transition. The HOMO−2 orbital is quite similar to the HOMO, but it displays a larger contribution of the thiophene ring and less conjugation along the π system. Finally, given the very different structures optimized for Z and Z-twist, it is not surprising to find quite different spectra, though the general behavior (hypsochromic shift, lower intensity) is the same as for the E/Z comparison.

The UV/VIS measurements in this work being carried out at acidic pH, we adapted our calculations to the experimental conditions. The three molecules contain an amino side that is most likely protonated. We estimated the pKa values of such moiety by means of MolGpKa [17]. We obtained the same value of 5.1 for the pKa of compounds Aziothio-a, Azothio-b, and Azothio-c, confirming that the amino function is protonated. In addition to B3LYP/6-311G(d,p) calculations, we also carried out calculations at the PBE0-D3/def2tpvz and B3LYP/def2svp levels to provide results at the same level of calculation as those used in refs. [9,18], respectively, for similar azothio species.

The computed energies and enthalpies (relative to the E isomer) are reported in Table 3, where the B3LYP/6-311G(d,p), the B3LYP/def2svp, and the PBE0-D3/def2tzvp levels of theory are compared.

Both energies and enthalpies are fairly similar for the different levels considered, and the key features already discussed for the deprotonated species still hold. However, we note that, in the protonated species, the E-twist conformer is more stable than (or as stable as) the E structure. A visual inspection reveals that one of the H atoms of the -${\mathrm{NH}}_{3}^{+}$ moiety points toward the N-N bond, possibly revealing some stabilizing Lewis acid/base interaction.

**Figure 4:**
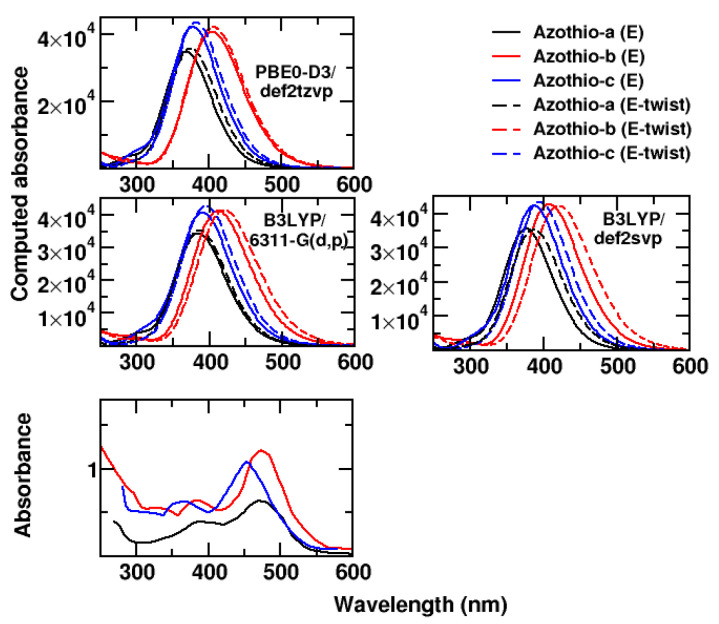
Comparison for spectra of protonated species. In the top and middle panels, we compare the computed spectra for the E and E-twist minima at three different levels of quantum chemistry. In the bottom panel, we report the experimental spectra: Azothio-a, black line; Azothio-b, red line; Azothio-c, blue line. In the computed spectra, those obtained for the E-twist conformations are reported as dashed lines.

In Figure 4, we report a comparison between the spectra obtained at the three levels of quantum chemistry considered (focusing on the most stable E isomers), as well as with the experimental results.

The different theoretical methods provide very similar results for the spectra of the E and of the E-twist minima. They predict a bathochromic shift for the two molecules bearing substituents, whereas the experimental measurements present very similar peaks, with the one of Azothio-c slightly blue-shifted. The PBE0/def2tzvp bands are blue-shifted with respect to the B3LYP results, whereas the B3LYP results obtained with the two different basis sets are very similar. As in the experiment, calculations predict small substituent effects on the band position.

We now focus on the B3LYP/6-311G(d,p) results for a quantitative comparison. The computed energies of the transition corresponding to the absorption maximum are 3.22, 2.95, and 3.13 eV for Azothio-a, -b, and -c, respectively, to be compared with 2.62, 2.61, and 2.74 eV, the corresponding experimental values. We thus obtain a relative error ranging between 13% and 23%, which is not satisfying, though consistent with the error in the evaluation of transition energies in aromatic systems [19,20], comprising thiophene azo dyes [21], if no scaling factor or correction is introduced [22].

Considering that a better qualitative agreement was obtained in the case of the neutral compounds, and that a change in the DFT functional/basis set did not produce any drastic change in our results, we may conjecture that the discrepancies seen with respect to experiments (both on the bands’ position and shape) are mainly due to the limitations of using a continuum representation of the solvent. This approximation is more drastic in the presence of the protonated molecules, which are charged and more exposed to strong intermolecular interactions with the real solvent, the DMSO–water mixture, which can both behave as hydrogen bond (H-bond) acceptors for the -${\mathrm{NH}}_{3}^{+}$ group. As some of us have already shown for the optical properties of a bipyridyl-diol in water, continuum models fail to deliver reliable UV/VIS spectra when stable solute–solvent hydrogen bonds are present in the system [23]. This consideration has motivated the following analysis, carried out to achieve a clear description of the solvation structure around each of the three molecules.

**Figure 5:**
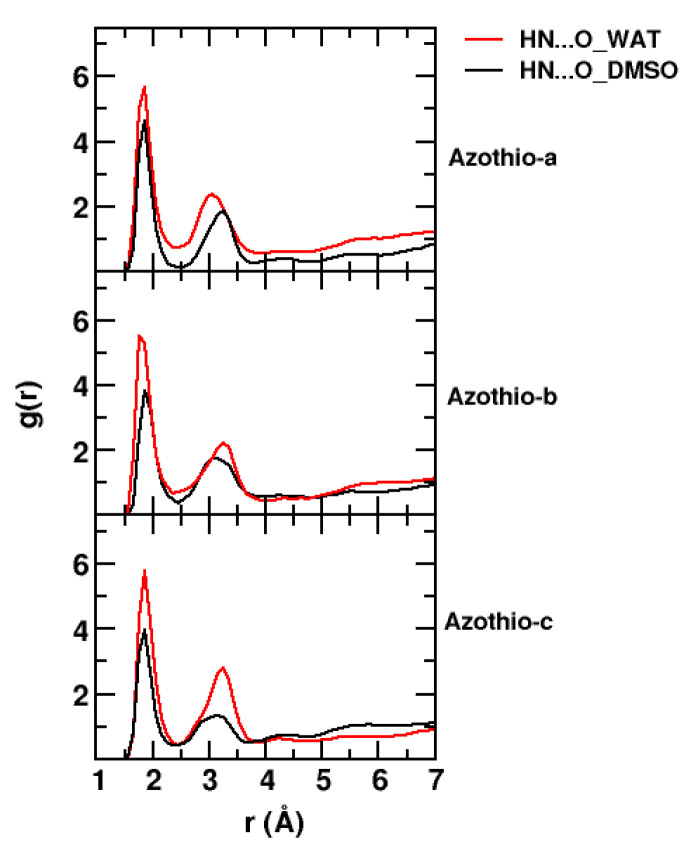
Radial distribution functions describing specific solute–solvent interactions for Azothio-a, Azothio-b and Azothio-c (from top to bottom), computed along the classical MD trajectories. The interactions between the H atoms on the protonated amino site of the solutes and (i) the O atoms of the water molecules (ii) the O atoms of DMSO molecules are displayed in red and in black, respectively.

Using classical molecular dynamics simulations, we characterized the local solvation structure of Azothio-a, -b, and -c in their protonated state and immersed in a mixture of DMSO and water. We computed the atom–atom radial distribution functions for all combinations of the relevant site. As we expected, the most remarkable result was obtained for the interactions between the H atoms of the amino site of the solute, the O atom of DMSO, and of water. Results are depicted in Figure 5.

A strong peak, located in all cases at about 1.8 Å, is associated with a strong intermolecular hydrogen bond for first neighbors. It is slightly broader and higher in the radial distributions involving water. A second, well-structured peak is also observed, the position of which is subject to some changes, depending on the system and the type of interaction. In the case of water, this second peak is slightly larger, higher, and situated at slightly larger distances (except for Azothio-a) compared with the second peak of the radial distribution involving DMSO. However, such differences may be attributed to the different sizes and shapes of water molecules compared with DMSO. Overall, we have compelling evidence of a robust and well-structured hydrogen bond network involving the solute and both components of the mixture.

**Figure 6:**
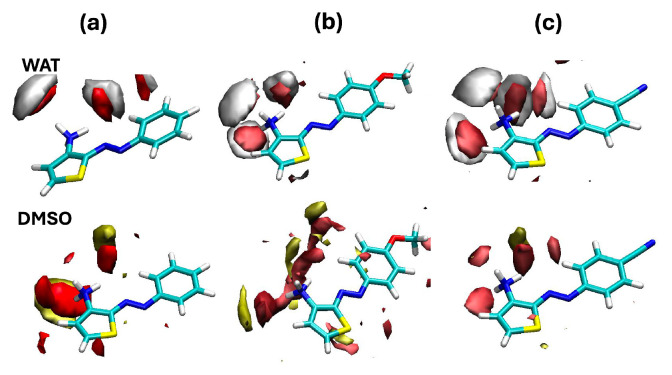
Three-dimensional histograms collecting the number density of significant solvent atoms surrounding (**a**) Azothio-a, (**b**) Azothio-b, and (**c**) Azothio-c along the classical MD trajectories. The O and the H atoms of water molecules are displayed in red and light gray, respectively, whereas the O and the S atoms of DMSO molecules are shown in red and yellow. Concerning the solute (licorice representation), C atoms are displayed in light blue, H atoms in white, O atoms in red, N atoms in blue, S atoms in yellow.

Radial distribution functions are very useful for identifying strong interactions, but they are complex to interpret when one seeks a 3-dimensional picture of the solvation shell around a nonspherical solute. We thus computed a 3D distribution of the number densities of the relevant solvent sites around the solute (generally referred to as spatial distribution functions), displayed in Figure 6.

We now clearly see that the amino group is strongly involved in interactions with water. The local water density around this moiety increases, going from Azothio-a to Azothio-c, whereas that of DMSO seems to decrease. In all cases, solvent atoms in the first solvation shell, the radius of which we can estimate to be about 2.5 Å from radial distribution functions, are tightly bound to the solutes, thus corroborating the hypothesis according to which specific interactions might play an impact on the properties of such molecules.

The following step in our work consisted in investigating the evolution of the system along 10 ps QM/MM dynamics. First of all, we focus on the solutes’ structural properties. The C-N-N-C dihedral is not subject to changes along the dynamics compared with the optimized structure obtained with B3LYP/6-311G(d,p) calculations with an implicit solvent. However, the N-N-C-S dihedral is slightly distorted, on average, compared with the static configuration, and fairly large fluctuations around the average value are observed. In the static case, we measured a value of 0.0° for all solutes, whereas in the QM/MM simulations, we found the following average values for Azothio-a to -c (standard deviations are reported in parentheses): −0.1° (±10°), 0.6° (±15°), −3° (±15°).

**Figure 7:**
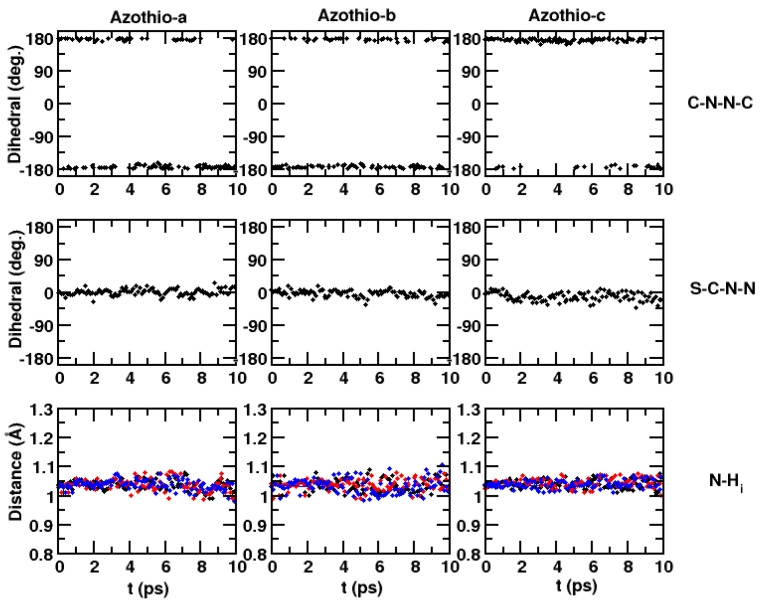
Time evolution of relevant geometric parameters in Azothio-a (**left**, black dots), Azothio-b (**center**, black dots), and Azothio-c (**right**) along the QM/MM dynamics. From top to bottom: C-N-N-C dihedral angle, S-C-N-N dihedral angle, distances between the N atom and each of the H atoms of the amino group (black, red and blue dots).

Another difference was found in the average N-H distance for the three acidic hydrogen atoms of the amino group. In the static case, all three molecules had an average N-H distance of 1.025 Å, while this average value increases to 1.040 Å (±0.03 Å) in the dynamics. Although the differences are small, these larger bond lengths are consistent with having the H atoms involved in hydrogen bonds with the surrounding environment. To illustrate such geometrical properties, we show in Figure 7 their time evolution along the simulated QM/MM trajectory.

**Figure 8:**
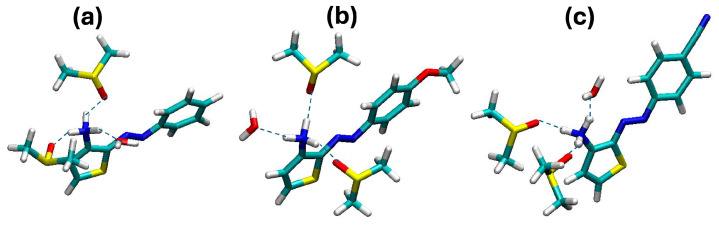
Water and DMSO molecules surrounding the amino group in (**a**) Azothio-a, (**b**) Azothio-b, and (**c**) Azothio-c, along the QM/MM dynamics. The directions of the hydrogen bonds are displayed as dashed lines, as a guide for the eye. The atoms colors are the same as defined in Figure 6.

Guided by the results obtained in the study of the solvation shell from classical MD, we finally analyzed the QM/MM trajectories by identifying the water and DMSO molecules around the amino site within a distance consistent with the size of the first solvation shell. In all cases, we found two DMSO molecules and one water molecule strongly bound to the amino site. An illustration of the solvation shell structure is reported in Figure 8. Along the 10 ps trajectories, the same DMSO and water molecules were present next to the amino groups, thus contributing to stabilizing solute–solvent-specific interactions.

For each of the three chromophores, we extracted one snapshot including the three solvent molecules for further analysis. We reoptimized such clusters using the B3LYP/6-311G(d,p) level in a PCM continuum and computed the absorption spectra based on the optimized structure. The new transition energies thus obtained were 3.05, 2.67, and 3.02 eV for Azothio-a, -b, -c, respectively. The relative error compared with the experiment was thus improved to 16%, 2%, and 10% (Azothio-a to -c) compared with the results that we obtained for the chromophores in a continuum. We interpret the fact that such improvement is not the same for all molecules as a sign of the need for a statistical distribution of structures extracted from MD. Nonetheless, such findings confirm our original hypothesis, according to which the local H-bond network around the charged groups is quite strong. We can safely conclude that a fine description of the optical properties of these species must explicitly include the surrounding solvation shell. However, including both explicit solvent molecules and finite temperature effects for the calculation of UV/VIS spectra and a fine description of both the band position and shape would require sophisticated, sequential methodologies that are beyond the scope of the present study [19,23,24,25,26,27,28].

## 3. Materials and Methods

### 3.1. Experimental Procedure

**Figure 9:**
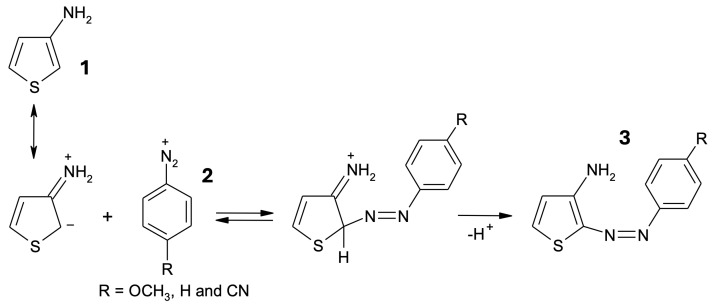
Proposed mechanistic pathway for the azo-coupling reactions of 3-aminothiophene with 4-R-benzenediazonium cations (R = OCH_3_, H, and CN) in a 50:50 water/DMSO (*v*/*v*) mixture at 298 K.

Because of the poor solubility of 3-aminothiophene (molecule **1** in Figure 9) in aqueous solution, the azo-coupling reactions of para-R-benzenediazonium cations (R = H, OCH_3_ and CN) with this compound were carried out in a 50:50 (*v*/*v*) H_2_O-DMSO mixture. 3-aminothiophene used in this study was prepared and characterized as previously described [29]. Experiments were performed by mixing HCl solutions ([H^+^] = 10^−3^ to 10^−1^ mol L^−1^) of diazonium cation (structures **2** in Figure 9, 5 × 10^−5^ mol L^−1^) with a large excess of 3-aminothiophene (2.20 × 10^−2^ to 1.75 × 10^−3^ mol L^−1^) at 298 K. The three 4-R-substituted diazonium cations were readily prepared from the addition of 4-R-substituted primary aromatic amines to a warm mixture of H_2_SO_4_ in water, as reported previously [15]. Dimethyl sulfoxide of the highest quality was available and used without further purification. The DMSO/water mixtures were prepared using the volumetric method. Under these experimental conditions, azothiophenes **3** (C-attack) were the only species formed, according to the scheme in Figure 9.

In line with this mechanism, Terrier and co-workers have observed selective C-attack in the reactions of 3-aminothiophene with 4,6-dinitrobenzofuroxan in the same water/DMSO mixture [30]. As in other reports in the literature, our results support the idea that 3-aminothiophene (pKaNH_2_ = 3.38 under these experimental conditions) exhibits an especially high enaminic character [15,30,31,32]. The electronic UV/VIS spectra were performed using a conventional spectrophotometer (Shimadzu UV–visible, Model 1650, Kyoto, Japan).

**Figure 10:**
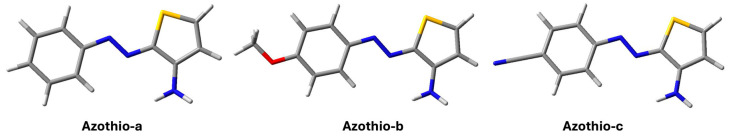
Names for the three compounds studied in this work (the E isomer is depicted in the three cases). H atoms are displayed in white, C atoms in gray, N atoms in blue, O atoms in red, S atoms in yellow.

From now on, we refer to the three azo-coupling products **3** in Figure 9 as Azothio-a (R = H), Azothio-b (R = OCH_3_), and Azothio-c (R = -CN), as summarized in Figure 10.

### 3.2. Simulations

Electronic structure calculations were carried out using Gaussian16 [33] and Gaussview6 [34] for part of the analysis and for creating images of the molecular structures. We performed a full optimization on the minima found along the potential energy surface and characterized them through an analysis of the frequencies of the normal modes (at 298 K and 1 bar). The stationary points were considered as minima only when no imaginary frequency was found. For the evaluation of the isomerization barriers (from E to Z and from Z to E), we optimized the transition states (TS), characterized by the presence of one imaginary frequency. All the optimized structures are available in the Supplementary Materials.

**Figure 11:**
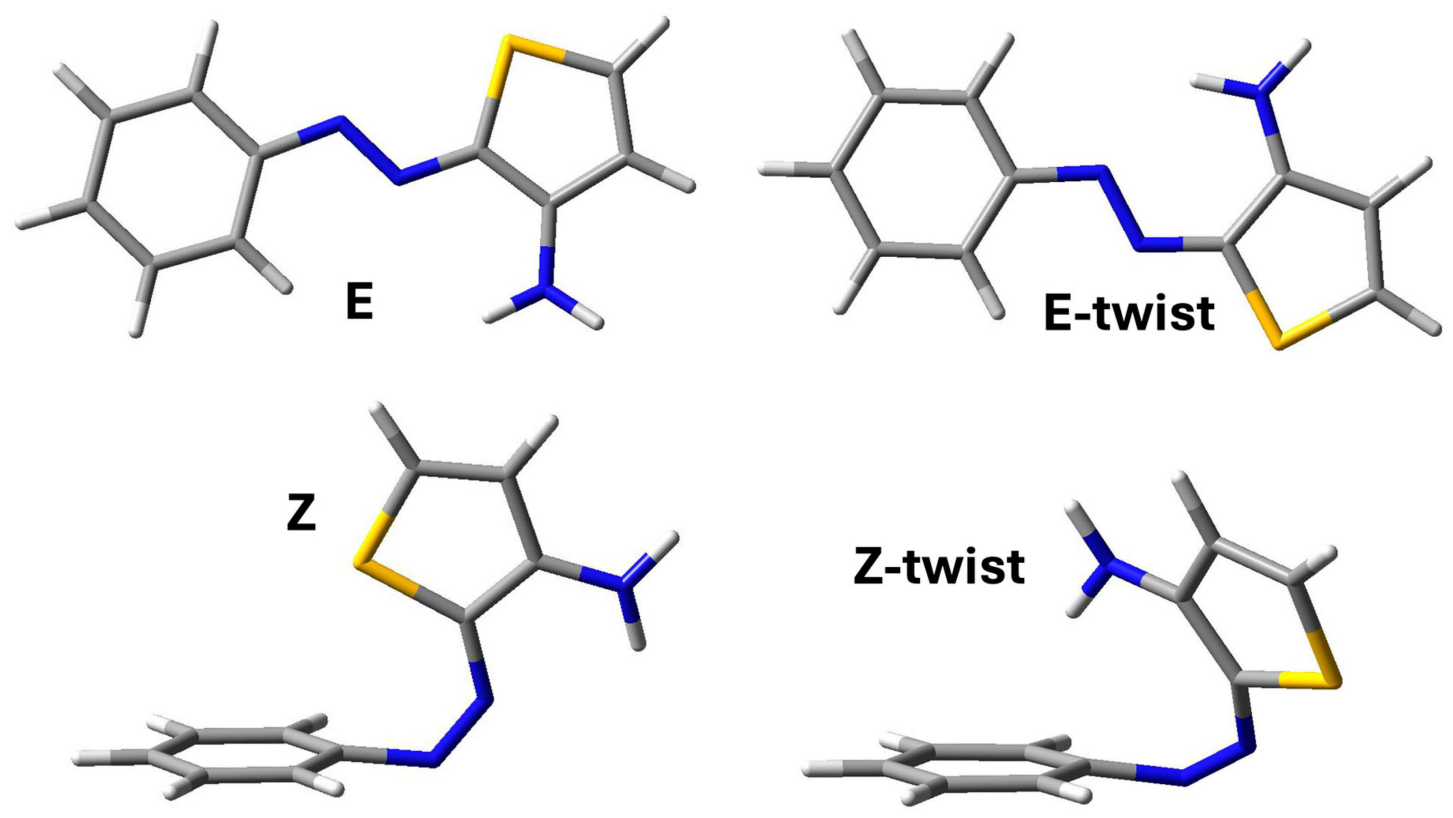
PES minima for the E and the Z isomers of Azothio-a.

The potential energy surface search for minima in the case of the E and Z isomers provided two additional structures, depicted in Figure 11, which we named ‘E-twist’ and ‘Z-twist’. This is fully consistent with literature reports on similar compounds [9,10,13]. In the case of Azothio-b, we conducted an additional scan along the dihedral angle defining the orientation of the methoxy group relative to the ring to ensure that the global minimum was accurately identified.

DFT calculations were carried out with the B3LYP [35] functional and the 6-311G(d,p) basis set [36,37], representing a reasonable compromise between accuracy and computational cost (see, for instance, refs. [18,21,38,39] for the use of B3LYP with azothiophenes). In the case of the protonated species, such results were compared with those obtained with the PBE0 functional, an empirical dispersion correction [40] and the def2TZVP basis set [41], and with the B3YLP functional using the def2SVP basis set [41]. These two quantum chemistry levels correspond with those employed in published studies of the absorption properties of azothiophenes [9,10,18]. We refer to these levels of theory as B3LYP/6-311G(d,p), PBE0-D3/def2tzvp, B3LYP/def2svp. Calculations were carried out using the polarizable continuum model (PCM) [42], with a value of the dielectric constant corresponding to that of the water/DMSO mixture at the experimental conditions [43].

Classical MD and hybrid QM/MM MD simulations were conducted using Amber16 [44] and visualized by means of VMD [45]. Classical MD simulations were carried out for Azothio-a, -b, and -c in a mixture of water and DMSO. The number of molecules and the box size were adjusted to be consistent with the density values reported in ref. [46] for a 50:50 (*v*/*v*) mixture. For water and DMSO molecules, we used the forced fields reported in that work, whereas we applied the Antechamber procedure in the AMBER16 suite of codes to parametrize the solutes, using the generalized Amber FF [47] for intramolecular and Lennard-Jones parameters. Periodic boundary conditions were taken into account with the particle mesh Ewald method [48] for treating long-range electrostatic, whereas Lennard-Jones intermolecular interactions were cut off at half of the simulation box. After minimization using the steepest descent algorithm, we performed a 2 ns equilibration in the NVT ensemble using Berendsen’s thermostat [49] at 298 K with a 1 fs time step. The production trajectory used for analysis was 2 ns long (same time step) in the NVT ensemble (298 K).

Additional B3LYP/6-31G(d,p)/MM MD simulations with the sander/Gaussian16 coupling scheme in AMBER [50] were carried out for the three cations surrounded by the water/DMSO mixture. For each simulation, starting configurations were taken from classical MD after equilibration (one solute molecule surrounded by 256 water molecules and 256 DMSO molecules). During such equilibration, the geometry of the solutes was constrained to the optimized geometry of the most stable structure at the B3LYP/6-311G(d,p) level in a continuum solvation medium. After minimization using the steepest descent algorithm, we performed a 100 fs equilibration in the NVT ensemble using Berendsen’s thermostat [49] at 298 K. The time step used in the simulations was 1 fs and the cut-off for Coulomb and Lennard-Jones interactions was fixed at 13 Å. After equilibration, each trajectory was propagated in the NVT ensemble (298 K) for 10 ps.

Data analysis for MD and QM/MM MD simulations was conducted employing postprocessing tools built in the AMBER16 package, as well as in-house codes.

## 4. Conclusions

Alternative systems to traditional azobenzene dyes are gaining increasing attention due to their broader range of structural and optical properties. These properties are valuable for applications in the textile industry as dyes and in technologies that utilize their photoswitching capabilities [5,6,7,8,9,10,18,39]. The structure-to-property relation for these molecules is key to advance in a rational design of photoactive systems, since it has been pointed out that an empirical relationship holds between the position of the absorption maximum of the electronic spectrum and the lifetime of the metastable Z isomers, an important factor for pharmaceutical applications [39]. Furthermore, molecules belonging to the family of azothiophene generally present a red-shifted absorption maximum compared with azobenzenes, which is important to avoid photodamage in biological systems, and well-separated absorption bands for the Z and E isomers, to optimize the photoconversion process.

Previous reports have extensively treated the effect of the presence of substituents of different natures on benzyl and thiophene rings [9,10,39]. One study has taken into account the protonation state of a substituted azothiophene displaying an -OH group [18]. However, to the best of our knowledge, explicit solvation effects have not been included in prior studies. In this work, we investigate newly conceived dyes obtained through an azo-coupling reaction of 3-aminothiophene with para-R-benzenediazonium cations (R = H, OCH_3_, and CN) in a mixture of water and dimethyl sulfoxide. We provided some insights on the potential energy surface of the neutral compounds in a continuum solvent and of the UV/VIS spectra. Such results are in very good agreement with those published for similar neutral systems [9]. Since the three molecules of interest display a protonated amino group under the experimental conditions, we carried out quantum chemistry calculations for the protonated species in a continuum solvent at two different levels of theory. The results thus obtained are in overall agreement, the only difference being a stabilizing effect of the E-twist structure with respect to E when using PBE0-D3/def2tzvp. However, with both levels, the computed UV/VIS spectra of the two species are very similar, and the bands, compared with the experimental ones, result more narrowly and blue-shifted. Considering that, notwithstanding the level of quantum chemistry, such discrepancy is present, we attributed it to the limited description of local solvation effects, particularly in the surrounding of the protonated amino group that can behave as a hydrogen bond donor.

To investigate this hypothesis further, we first run classical MD simulations of the three cations in a mixture of water and DMSO, carefully incorporating the experimental conditions. From such simulations, we were able to draw a clear picture of the solvation shell, comprising water and DMSO molecules H-bonded to the amino site. The systems were then treated at the B3LYP/6-31G(d,p)/MM level on a 10 ps timescale, during which only minor changes in the geometry of the E isomer were observed, and the solvation shell surrounding the protonated amino group did not evolve, displaying two DMSO molecules and one water molecule strongly H-bonded to the acidic H atoms. Absorption spectra computed on the clusters structures extracted from such simulations, reoptimized at the B3LYP/6-311G(d,p) level in a continuum, provided an improved agreement with the experimental transition energies.

Our results highlight the importance of including explicit solvent molecules when modeling the structural and optical properties of substituted azothiophenes, as these molecules play a crucial role in the solvation environment. Future research will extend this work by incorporating explicit solvation into the study of the complete potential energy surface, including the Z/E photoconversion steps, and by enhancing the accuracy of UV/VIS spectra predictions.

## Figures and Tables

**Table 1:** Relative energies and standard enthalpies at 298 K for the PES minima found for Azothio-a, -b, and -c, evaluated at the B3LYP/6-311(d,p) level. For each molecule, values are reported with respect to the corresponding ones for the global minimum (E isomer).

Molecule	ΔE kJ/mol	ΔH kJ/mol
Azothio-a (E)	0.0	0.0
Azothio-a (E-twist)	7.0	6.6
Azothio-a (Z)	58.2	57.1
Azothio-a (Z-twist)	86.6	84.8
Azothio-b (E)	0.0	0.0
Azothio-b (E-twist)	7.9	7.1
Azothio-b (Z)	61.7	60.1
Azothio-b (Z-twist)	87.7	85.9
Azothio-c (E)	0.0	0.0
Azothio-c (E-twist)	6.6	6.3
Azothio-c (Z)	60.4	59.1
Azothio-c (Z-twist)	90.3	88.5

**Table 2:** Barriers for the E to Z (E/Z) and the Z to E (Z/E) conversions, in terms of standard activation energies at 298 K (for which the ^‡^ symbol is used) for Azothio-a, -b, and -c, evaluated at the B3LYP/6-311(d,p) level.

Molecule	ΔG ^‡^ E/Z kJ/mol	ΔG ^‡^ Z/E kJ/mol
Azothio-a	146.1	89.3
Azothio-b	160.9	101.2
Azothio-c	120.2	59.8

**Table 3:** Relative energies and standard enthalpies at 298 K for the PES minima found for protonated Azothio-a, -b, and -c, evaluated at the B3LYP/6-311(d,p), B3LYP/def2svp, and PBE0-D3/def2tzvp levels. For each molecule, values are reported with respect to the corresponding ones for the E isomer.

Molecule	ΔE kJ/mol	ΔH kJ/mol	ΔE kJ/mol	ΔH kJ/mol	ΔE kJ/mol	ΔH kJ/mol
	B3LYP/6-311G(d,p)		B3LYP/def2svp		PBE0-D3/def2tzvp	
Azothio-a (E)	0.0	0.0	0.0	0.0	0.0	0.0
Azothio-a (E-twist)	2.2	0.7	−2.9	−4.7	−0.7	−2.5
Azothio-a (Z)	56.7	54.8	57.6	56.4	54.3	51.5
Azothio-a (Z-twist)	92.5	91.4	90.5	88.6	70.1	69.4
Azothio-b (E)	0.0	0.0	0.0	0.0	0.0	0.0
Azothio-b (E-twist)	1.5	0.1	−3.2	−4.8	−8.0	−7.3
Azothio-b (Z)	59.3	58.1	65.1	63.3	47.8	49.4
Azothio-b (Z-twist)	88.9	86.8	93.5	92.1	66.7	67.9
Azothio-c (E)	0.0	0.0	0.0	0.0	0.0	0.0
Azothio-c (E-twist)	1.4	0.1	−2.0	−4.2	−0.7	−2.5
Azothio-c (Z)	67.3	65.5	54.6	52.8	54.3	51.5
Azothio-c (Z-twist)	92.0	90.4	93.7	92.8	70.1	69.4

## Data Availability

The optimized structures of the minima (E, Z, E-twist, Z-twist isomers of Azothio-a, -b, and -c) and of the transition states (E to Z isomerization) are provided as Supplementary Materials.

## Supplementary Materials

The following supporting information can be downloaded at https://www.mdpi.com/article/10.3390/molecules29174053/s1: optimized geometries obtained at different levels of theory for the three molecules Azothio-a, -b and -c in their deprotonated and protonated states. Minima are reported as well as the transition state structure for the E to Z isomerization.
