# Supplementary material for: Structural and Electronic Properties of Novel Azothiophene Dyes: A Multilevel Study Incorporating Explicit Solvation Effects
Source: Molecules. 2024 Aug 27;29(17):4053. doi: 10.3390/molecules29174053 (PMC11397383; doi:10.3390/molecules29174053)
Supplement: Supplementary file 1 [file molecules-29-04053-s001.zip › molecules-3158718-supplementary.pdf]

Supporting Information

Structural and Electronic Properties of Novel Azothiophene Dyes. A Multilevel Study Incorporating Explicit Solvation Effects.

Laura Vautrin, Alexandrine Lambert, Faouzi Mahdhaoui, Riad El Abed, Taoufik Boubaker, Francesca Ingrosso

A Optimized Geometries

In the following pages, we provide the optimized geometries obtained at different levels of theory for the three molecules Azothio-a, -b and -c in their deprotonated and protonated states. Minima are reported as well as the transition state structure for the E to Z isomerization.

DEPROTONATED SPECIES

E isomers, B3LYP/6-311G(d,p)

Azothio-c

C	-4.57878100	-0.79954900	0.00030000
C	-4.57474500	0.56099800	0.00009400
C	-3.25146300	1.10860400	-0.00011700
C	-2.26708200	0.09435900	-0.00008400
S	-3.00402600	-1.53023900	0.00025500
H	-5.45011200	-1.43790100	0.00049000
H	-5.47088600	1.16610100	0.00011100
N	-2.97151800	2.42215700	-0.00028800
H	-3.70223500	3.11397400	-0.00068500
H	-2.01334300	2.73317300	-0.00050700
N	-0.95707500	0.34157900	-0.00029300
N	-0.16938400	-0.67030600	-0.00024200
C	1.19558200	-0.35459000	-0.00012500
C	2.07893800	-1.44839000	-0.00041400
C	1.72660100	0.95100300	0.00027700
C	3.44992600	-1.25608800	-0.00033900
H	1.66177900	-2.44788500	-0.00070900
C	3.09467800	1.14930900	0.00036600
H	1.05165100	1.79554000	0.00051900
C	3.97173800	0.04818400	0.00005200
H	4.12082900	-2.10592100	-0.00057900
H	3.49817200	2.15447700	0.00068400
C	5.38238400	0.25710500	0.00013800
N	6.52682200	0.42751400	0.00020800

Azothio-a

C	-3.85954900	-0.92878600	0.01650000
C	-3.91499000	0.43107800	0.01014100
C	-2.61822300	1.03911900	-0.00578300
C	-1.59347400	0.07921700	-0.00614200
S	-2.24919100	-1.57717600	0.00127500
H	-4.69833700	-1.60897300	0.02804800
H	-4.83719600	0.99643300	0.01334200
N	-2.40689500	2.37784700	-0.06082300
H	-3.15582600	3.00341300	0.19008700
H	-1.46885100	2.71516300	0.09192500
N	-0.28426800	0.38688100	-0.00442300
N	0.53820000	-0.58625000	-0.01053300
C	1.90174200	-0.22672200	-0.00320700
C	2.81412400	-1.29224200	-0.00731200
C	2.39040200	1.09188300	0.00649400
C	4.18458700	-1.05104800	-0.00184700
H	2.42428000	-2.30358100	-0.01498300
C	3.75956300	1.32523700	0.01149900
H	1.68805700	1.91476300	0.00949700
C	4.66389400	0.25860700	0.00749000
H	4.87790400	-1.88447500	-0.00503100
H	4.12850600	2.34504900	0.01862300

H	5.73079600	0.44963100	0.01155900

Azothio-b

C	-4.72070700	-0.95801200	0.02291000
C	-4.78652000	0.40159800	0.01190300
C	-3.49530900	1.02111000	-0.00868900
C	-2.46367300	0.07431100	-0.00718000
S	-3.10238100	-1.58748400	0.00590300
H	-5.55207200	-1.64699000	0.03858000
H	-5.71351400	0.95942000	0.01444700
N	-3.29779800	2.36832800	-0.07784700
H	-4.04304300	2.97380000	0.22985200
H	-2.36358200	2.70329000	0.10481800
N	-1.15219600	0.39493900	-0.00673700
N	-0.32509700	-0.57257700	-0.01386700
C	1.03448000	-0.21408400	-0.00598200
C	1.95314900	-1.26949900	-0.01604600
C	1.52855700	1.10595800	0.01028800
C	3.32639400	-1.03926800	-0.01064600
H	1.57227600	-2.28414300	-0.02861600
C	2.88895100	1.34405900	0.01548400
H	0.82947400	1.93160800	0.01837700
C	3.80379300	0.27541700	0.00511000
H	4.00573600	-1.88032700	-0.01886200
H	3.27887200	2.35522000	0.02780800
O	5.11764700	0.62311400	0.01161100
C	6.09979300	-0.41521100	0.00212700
H	7.06337300	0.08983800	0.00958500
H	6.01402100	-1.04983600	0.88909000
H	6.01757800	-1.03016200	-0.89891900

E-twist isomers, B3LYP/6-311G(d,p)

Azothio-c

C	4.73843000	-0.57664800	0.00017000
C	4.42309100	0.75005200	0.00013200
C	3.01388500	0.98702000	0.00008100
C	2.27799300	-0.22799700	0.00008900
S	3.36687000	-1.62672000	0.00019200
H	5.72991600	-1.00436600	0.00020300
H	5.15990100	1.54211400	0.00012900
N	2.42894600	2.19484000	-0.00000400
H	2.97795500	3.03945700	0.00019900
H	1.41607800	2.22418300	0.00009200
N	0.95832100	-0.45628000	0.00007200
N	0.18659700	0.57709000	-0.00004700
C	-1.18314400	0.27710400	-0.00007300
C	-2.05705900	1.37825200	-0.00027900
C	-1.72451100	-1.02383900	0.00010600
C	-3.42967700	1.19699700	-0.00031600
H	-1.63537300	2.37601800	-0.00041200
C	-3.09429700	-1.21068800	0.00007200
H	-1.05510800	-1.87254600	0.00027300

C	-3.96245000	-0.10263800	-0.00014100
H	-4.09339500	2.05246300	-0.00047900
H	-3.50588600	-2.21257300	0.00021200
C	-5.37485800	-0.29936100	-0.00017500
N	-6.52078200	-0.45947200	-0.00020300

Azothio-a

C	-4.08392200	-0.45022000	0.00003600
C	-3.70930600	0.86107200	-0.00027300
C	-2.28982200	1.03800600	-0.00023900
C	-1.60973100	-0.20106200	0.00011200
S	-2.75355400	-1.55248100	0.00042600
H	-5.09201800	-0.83661900	0.00011100
H	-4.41134500	1.68442900	-0.00052600
N	-1.65542700	2.22668000	-0.00056600
H	-2.16998700	3.09204900	-0.00077400
H	-0.64323100	2.21538300	-0.00027800
N	-0.29249600	-0.49202500	0.00016500
N	0.51980200	0.50062000	0.00006600
C	1.88611500	0.14643300	0.00001800
C	2.79894200	1.21163400	0.00059300
C	2.37555600	-1.17179800	-0.00062200
C	4.16956700	0.97003300	0.00058100
H	2.41337700	2.22487900	0.00106900
C	3.74488300	-1.40519300	-0.00064100
H	1.67220700	-1.99358500	-0.00110500
C	4.64975600	-0.33925700	-0.00003200
H	4.86257400	1.80382200	0.00104800
H	4.11320000	-2.42532900	-0.00115100
H	5.71670300	-0.53013200	-0.00005800

Azothio-b

C	-4.95222100	-0.44789200	0.01969100
C	-4.57607200	0.86311400	0.02322300
C	-3.15661000	1.04107600	0.00191000
C	-2.47706800	-0.19408600	-0.01287400
S	-3.61825500	-1.54610500	-0.00777600
H	-5.95945800	-0.83606300	0.02979100
H	-5.27772900	1.68689700	0.03784000
N	-2.52598900	2.23725600	-0.02211900
H	-3.04162500	3.09050200	0.11989000
H	-1.51636800	2.22696500	0.04419100
N	-1.15579900	-0.48983200	-0.01369800
N	-0.34440900	0.50138500	-0.01815800
C	1.02033000	0.15387600	-0.00947000
C	1.93501500	1.21244600	-0.01445300
C	1.52038700	-1.16359100	0.00291500
C	3.30970700	0.98714600	-0.00742200
H	1.55439500	2.22745800	-0.02436000
C	2.88209700	-1.39647100	0.00957300
H	0.82356600	-1.99095100	0.00688800
C	3.79291000	-0.32507100	0.00465600

H	3.98540300	1.83127400	-0.01151800
H	3.27520400	-2.40650500	0.01894200
O	5.10923300	-0.66772000	0.01230700
C	6.08606500	0.37507100	0.00864900
H	7.05225600	-0.12510000	0.01641000
H	6.00311900	0.99316900	-0.89030200
H	5.99560200	1.00641900	0.89761000

Z isomers, B3LYP/6-311G(d,p)

Azothio-c

C	2.51116900	2.15363500	-0.26658300
C	3.63535600	1.41300700	-0.06865300
C	3.35520800	0.02172500	0.09710600
C	1.97220300	-0.26603200	0.02408300
S	1.03905500	1.24492400	-0.24670100
H	2.46103200	3.22216800	-0.41859700
H	4.63333600	1.82866100	-0.04268400
N	4.29514500	-0.91916100	0.32470800
H	4.00727100	-1.88606400	0.29554600
H	5.26554300	-0.71410200	0.14999300
N	1.57814400	-1.55731400	0.05456900
N	0.41066800	-2.04149500	-0.02506200
C	-0.76183700	-1.23773300	-0.01256700
C	-1.57774600	-1.20410500	-1.15290700
C	-1.20825200	-0.63915500	1.17677000
C	-2.79959300	-0.55101700	-1.12097100
H	-1.24064600	-1.69348000	-2.05845200
C	-2.43630200	0.00195400	1.21792800
H	-0.59332100	-0.69239900	2.06680400
C	-3.23765300	0.05902400	0.06613300
H	-3.42052700	-0.51387000	-2.00725500
H	-2.77954700	0.46036000	2.13695000
C	-4.50032400	0.72387200	0.10516000
N	-5.52272700	1.26352800	0.13606100

Azothio-a

C	2.19177700	1.98526300	-0.14108900
C	3.21182800	1.08864200	-0.04418700
C	2.75173400	-0.26091000	0.04181900
C	1.34475000	-0.35844200	0.00690400
S	0.61447500	1.27604800	-0.11984800
H	2.28091900	3.05926900	-0.21879500
H	4.25775900	1.36381700	-0.03358200
N	3.56968100	-1.33254600	0.18070000
H	3.15037500	-2.24417100	0.06979300
H	4.54095500	-1.24363000	-0.07206700
N	0.78218100	-1.59305600	-0.00989400
N	-0.43966700	-1.91613600	-0.05525300
C	-1.48140300	-0.93191100	-0.00834200
C	-2.19990500	-0.63417400	-1.17197400
C	-1.89559000	-0.39758600	1.21834900
C	-3.29837300	0.22001600	-1.11250000

H	-1.89020700	-1.07453900	-2.11290200
C	-3.00201500	0.44686900	1.27061600
H	-1.35411900	-0.65333900	2.12205500
C	-3.70256400	0.76468300	0.10688500
H	-3.84332600	0.45510500	-2.01995000
H	-3.31751700	0.85637100	2.22378400
H	-4.56323500	1.42181900	0.15146700

Azothio-b

C	-2.57469400	2.13541200	-0.48996200
C	-3.70505200	1.46442600	-0.13624000
C	-3.45295900	0.09695300	0.19206900
C	-2.09035000	-0.24736900	0.08227700
S	-1.13214200	1.18066400	-0.42614000
H	-2.50323400	3.17267700	-0.78379300
H	-4.68882700	1.91313700	-0.10892100
N	-4.41022900	-0.77456000	0.60136600
H	-4.14467100	-1.74880500	0.63078100
H	-5.37233800	-0.57818600	0.37367800
N	-1.74250700	-1.55380400	0.22318000
N	-0.60223100	-2.09548000	0.13796500
C	0.59444600	-1.31657000	0.01538100
C	1.08037900	-0.55429000	1.08061500
C	1.39560000	-1.47232300	-1.12520400
C	2.32586500	0.06952300	1.00674700
H	0.48965800	-0.45709400	1.98429600
C	2.62432300	-0.83798700	-1.21520100
H	1.04039700	-2.09199800	-1.94060400
C	3.10112800	-0.05996800	-0.14976200
H	2.67622400	0.64574900	1.85217200
H	3.24123500	-0.94058100	-2.10014400
O	4.32543300	0.51032100	-0.33118700
C	4.86632000	1.30988200	0.72124100
H	5.83081300	1.66103600	0.36002200
H	4.22443400	2.16927700	0.93795100
H	5.01185300	0.72236100	1.63293300

Z-twist isomers, B3LYP/6-311G(d,p)

Azothio-c

C	-3.84781500	-1.32126900	0.17250700
C	-2.78328200	-1.76087100	-0.55348300
C	-1.73785300	-0.79253200	-0.68950900
C	-2.04329700	0.40439400	0.00228700
S	-3.67022000	0.30187000	0.71724500
H	-4.73445900	-1.88137000	0.42911800
H	-2.72305300	-2.74824100	-0.99232600
N	-0.63389100	-1.05276500	-1.43351200
H	0.02711400	-0.32754900	-1.65400700
H	-0.66703400	-1.82568800	-2.08036200
N	-1.51876800	1.66108500	0.08428700
N	-0.31323100	2.02639000	-0.07729800
C	0.80959300	1.17629700	-0.02405700

C	1.89972500	1.50558400	-0.85058700
C	0.96392000	0.16426600	0.94215600
C	3.09713400	0.81926500	-0.74879800
H	1.78377500	2.30830100	-1.56847300
C	2.17167900	-0.50144000	1.06941800
H	0.14389100	-0.08357200	1.60350300
C	3.24505900	-0.18974400	0.21867400
H	3.92413300	1.06640700	-1.40239100
H	2.28859000	-1.27008500	1.82306400
C	4.48233700	-0.88950700	0.34094700
N	5.48551500	-1.45682000	0.43918100

Azothio-a

C	-3.44619100	-0.89733300	0.24904800
C	-2.43550800	-1.60975000	-0.32014500
C	-1.24922400	-0.84322900	-0.56350000
C	-1.39256200	0.48744000	-0.11882500
S	-3.03637600	0.75654600	0.50120400
H	-4.41395500	-1.26617100	0.55373800
H	-2.51089000	-2.66024000	-0.57059700
N	-0.20061600	-1.36998300	-1.25675900
H	0.71675700	-0.96011800	-1.17405600
H	-0.21339700	-2.36308200	-1.43159700
N	-0.68969600	1.66165000	-0.25545600
N	0.56218500	1.81488900	-0.36274300
C	1.52469000	0.81295800	-0.06142900
C	2.66417400	0.74480200	-0.87822000
C	1.47651400	0.04649700	1.11535400
C	3.71510400	-0.10600100	-0.54929300
H	2.70586300	1.36606700	-1.76535900
C	2.54659100	-0.77701600	1.45202700
H	0.61441000	0.11197100	1.76752200
C	3.66352000	-0.86609700	0.61996300
H	4.58158800	-0.16330200	-1.19802700
H	2.50653200	-1.35367700	2.36914300
H	4.48932600	-1.51570900	0.88479300

Azothio-b

C	3.90390600	-1.41738600	0.06940900
C	2.88816100	-1.66741100	-0.80184500
C	1.88375300	-0.64580700	-0.83054600
C	2.16335300	0.38464700	0.08519400
S	3.71918700	0.08881800	0.88840200
H	4.74766900	-2.05275600	0.29201100
H	2.83399800	-2.55454100	-1.42016600
N	0.86892300	-0.66904300	-1.74783000
H	0.01435900	-0.17344900	-1.54201500
H	0.73980200	-1.53143800	-2.25565600
N	1.68315600	1.65784000	0.30877500
N	0.49668400	2.08068400	0.17548300
C	-0.65696300	1.26192500	0.06153400
C	-0.86535100	0.07857600	0.78575600

C	-1.73931400	1.80173300	-0.65977600
C	-2.10677700	-0.55316500	0.78509600
H	-0.06293100	-0.34434900	1.37680900
C	-2.96223500	1.15850800	-0.69792700
H	-1.59361000	2.73684700	-1.18780800
C	-3.16152500	-0.02524700	0.03133400
H	-2.23676900	-1.45262800	1.37091100
H	-3.78974900	1.56344300	-1.26785000
O	-4.40205700	-0.57048400	-0.05105400
C	-4.67714800	-1.76513700	0.68532400
H	-5.71514200	-2.01016000	0.47106000
H	-4.03373500	-2.58768800	0.36000500
H	-4.55491900	-1.60426600	1.76037000

TS, B3LYP/6-311G(d,p)

Azothio-a

C	2.92890300	1.61389100	0.37013900
C	3.57366200	0.42642200	0.55114900
C	2.77302200	-0.68503400	0.16321500
C	1.46748200	-0.26685900	-0.29702600
S	1.31613500	1.52607900	-0.26089100
H	3.34384300	2.58854000	0.58636600
H	4.58117500	0.33969700	0.93189400
N	3.13247700	-1.95766800	0.19969800
H	4.04795300	-2.24364600	0.51277000
H	2.47951500	-2.66649600	-0.10528600
N	0.55577600	-1.10329400	-0.68823500
N	-0.57890700	-0.67394700	-1.19748200
C	-1.69680500	-0.37683900	-0.49598400
C	-2.85445100	0.08337900	-1.18696300
C	-1.80533300	-0.53102500	0.91756200
C	-4.02639100	0.36882600	-0.50569300
H	-2.79757100	0.20572500	-2.26326000
C	-2.99704200	-0.24338600	1.57382700
H	-0.94696200	-0.88247500	1.47870200
C	-4.12262200	0.21203300	0.88405300
H	-4.88611600	0.72065200	-1.06849200
H	-3.04354100	-0.37970100	2.65069700
H	-5.04443300	0.43636400	1.40732100

Azothio-b

C	-3.60343200	1.77215600	-0.28429200
C	-4.28610800	0.66186500	-0.68428800
C	-3.57926900	-0.53899100	-0.39124900
C	-2.30380000	-0.26871700	0.23113100
S	-2.06102400	1.50066300	0.46116600
H	-3.94910900	2.79061100	-0.39423800
H	-5.25810100	0.68737300	-1.15596800
N	-3.99708400	-1.77022800	-0.63894800
H	-4.89252000	-1.95117500	-1.06671800
H	-3.41159000	-2.55363800	-0.38398900
N	-1.47297800	-1.20388800	0.57940700

N	-0.36914700	-0.91500300	1.23453300
C	0.82799100	-0.60849100	0.66631600
C	1.94783100	-0.32469200	1.48714000
C	1.04943900	-0.58619700	-0.74024300
C	3.19869700	-0.03633800	0.94819400
H	1.81817300	-0.33567600	2.56376800
C	2.30423900	-0.30427800	-1.26492100
H	0.22602600	-0.79895700	-1.41213400
C	3.39399100	-0.02290100	-0.43710100
H	4.01637300	0.17493200	1.62594700
H	2.45445800	-0.29931500	-2.33983600
O	4.59420200	0.24894900	-1.06889000
C	5.71958400	0.53499600	-0.25144100
H	6.55029800	0.71698000	-0.93222100
H	5.55790000	1.42785800	0.36400300
H	5.97034200	-0.30784400	0.40344300

Azothio-c

C	-3.39189100	1.80153300	0.04533000
C	-4.06708200	0.81100500	-0.60307200
C	-3.37186400	-0.42876600	-0.55979600
C	-2.10546800	-0.30931800	0.14366400
S	-1.86946500	1.36648400	0.75339800
H	-3.73065500	2.82302800	0.14903400
H	-5.02515600	0.94710200	-1.08335700
N	-3.77927900	-1.57223800	-1.07502500
H	-4.66453700	-1.65308200	-1.55340500
H	-3.20186400	-2.39714200	-0.97905900
N	-1.30092400	-1.30819500	0.29631400
N	-0.21020700	-1.20020600	1.03340100
C	0.97130300	-0.76182300	0.58935600
C	2.06988600	-0.68283700	1.50523800
C	1.23164500	-0.39724600	-0.77291600
C	3.31221700	-0.26833400	1.09481800
H	1.89645400	-0.95841200	2.53880400
C	2.48596600	0.00916900	-1.16994800
H	0.42908000	-0.45170100	-1.49812500
C	3.55795000	0.09084900	-0.25431500
H	4.12202900	-0.21470100	1.81389400
H	2.65456300	0.27222500	-2.20864500
C	4.84076900	0.51579500	-0.67190400
N	5.89482900	0.86475100	-1.01358100

E isomers, PBE0-D3/def2tzvp

Azothio-c

C	-4.54874200	-0.78878700	-0.00004600
C	-4.54304000	0.56999300	0.00005200
C	-3.22366200	1.10379400	0.00008800
C	-2.25701700	0.08081900	0.00000300
S	-2.99736700	-1.50724500	-0.00014000
H	-5.42524700	-1.42140700	-0.00008800
H	-5.43720500	1.17827300	0.00010800

N	-2.92996300	2.40744200	0.00011500
H	-3.65272700	3.10378900	0.00053700
H	-1.97026000	2.70627500	0.00043600
N	-0.94828200	0.32374700	0.00005000
N	-0.17095800	-0.67893200	-0.00001400
C	1.18707500	-0.36293300	-0.00000600
C	2.06758300	-1.44958200	0.00020100
C	1.70726000	0.93959500	-0.00023500
C	3.43246000	-1.25288400	0.00021300
H	1.65462200	-2.45128200	0.00035800
C	3.06895600	1.14184400	-0.00023200
H	1.02917800	1.78256900	-0.00042200
C	3.94307200	0.04765600	0.00000000
H	4.10841700	-2.09897300	0.00038600
H	3.47076000	2.14792500	-0.00041500
C	5.34907100	0.26178000	0.00000300
N	6.48952300	0.43645400	0.00000500

Azothio-a

C	-3.82803900	-0.92954200	0.01710000
C	-3.89110000	0.42818200	0.00896100
C	-2.60209400	1.03132000	-0.00746800
C	-1.58727000	0.06873100	-0.00665900
S	-2.23783700	-1.55433500	0.00374600
H	-4.66669600	-1.61085200	0.02950900
H	-4.81566800	0.98999800	0.01210600
N	-2.38638600	2.36199100	-0.06094600
H	-3.13485100	2.98938500	0.17573300
H	-1.44942200	2.69737700	0.08678800
N	-0.27984900	0.37643100	-0.00569200
N	0.53273300	-0.58818000	-0.00999400
C	1.88910200	-0.22949300	-0.00304900
C	2.79852100	-1.28790700	-0.01048400
C	2.36921400	1.08462700	0.01016100
C	4.16263400	-1.04376800	-0.00537800
H	2.41245900	-2.30109200	-0.02057500
C	3.73192400	1.32049200	0.01491400
H	1.66517600	1.90696800	0.01623600
C	4.63549000	0.26132500	0.00722100
H	4.85857700	-1.87498000	-0.01133700
H	4.09829300	2.34122300	0.02494100
H	5.70176000	0.45585600	0.01116900

Azothio-b

C	-4.68687300	-0.95199700	0.02289100
C	-4.75454100	0.40600200	0.01058000
C	-3.46837100	1.01480200	-0.01036000
C	-2.45075500	0.06093300	-0.00805000
S	-3.09134300	-1.56527500	0.00749100
H	-5.52182900	-1.63778300	0.03927400
H	-5.68116700	0.96469300	0.01365200
N	-3.25945200	2.35310600	-0.07755400

H	-4.00120000	2.96327600	0.22055800
H	-2.32482800	2.68066400	0.10414600
N	-1.14083400	0.38032900	-0.00791800
N	-0.32307800	-0.57775500	-0.01331800
C	1.02882600	-0.21900100	-0.00552600
C	1.94516100	-1.26654700	-0.01689000
C	1.51331500	1.09709200	0.01245000
C	3.31202700	-1.03272500	-0.01160700
H	1.56848300	-2.28328800	-0.03054800
C	2.86716400	1.33883200	0.01766000
H	0.81175000	1.92167900	0.02197400
C	3.78142500	0.27803500	0.00559700
H	3.99516600	-1.87108200	-0.02099500
H	3.25177000	2.35233000	0.03146800
O	5.08468400	0.62333900	0.01208900
C	6.04813700	-0.41257300	0.00038300
H	7.01944200	0.07762800	0.00785400
H	5.95723700	-1.04986900	0.88521700
H	5.95965600	-1.02744200	-0.90042700

E-twist isomers, PBE0-D3/def2tzvp

Azothio-c

C	4.70197000	-0.57993000	0.00024900
C	4.39423800	0.74709100	0.00011400
C	2.99395400	0.98157900	0.00001100
C	2.26859000	-0.23356700	0.00010300
S	3.34445000	-1.60474000	0.00020400
H	5.69434100	-1.00746100	0.00034200
H	5.13319900	1.53732600	0.00009200
N	2.40824100	2.18144500	-0.00015700
H	2.95491200	3.02441800	-0.00003500
H	1.39740500	2.20752100	-0.00013600
N	0.95161700	-0.46025600	0.00014300
N	0.19075300	0.56338000	0.00009000
C	-1.17288800	0.26895200	0.00000100
C	-2.03644000	1.36891100	-0.00018700
C	-1.71175600	-1.02526400	0.00011700
C	-3.40403700	1.19290900	-0.00027400
H	-1.61005800	2.36511200	-0.00026800
C	-3.07667000	-1.20670700	0.00003200
H	-1.04565900	-1.87753300	0.00027700
C	-3.93420800	-0.09975100	-0.00016600
H	-4.06694800	2.04922000	-0.00042500
H	-3.49349400	-2.20665500	0.00012300
C	-5.34340000	-0.29215200	-0.00025100
N	-6.48647000	-0.44864400	-0.00032100

Azothio-a

C	-4.05169300	-0.45503800	0.00020700
C	-3.68413100	0.85697200	-0.00038500
C	-2.27386700	1.03135500	-0.00043700
C	-1.60443000	-0.20792900	0.00011600

S	-2.73490900	-1.53126800	0.00071200
H	-5.06083900	-0.84071900	0.00037500
H	-4.38818500	1.67875200	-0.00075600
N	-1.63896700	2.21190000	-0.00094200
H	-2.15170200	3.07531500	-0.00164700
H	-0.62891800	2.19797100	-0.00090300
N	-0.29000400	-0.49726900	0.00026100
N	0.51085500	0.48665400	-0.00000800
C	1.87091800	0.14007200	-0.00001200
C	2.77219300	1.20549400	0.00102900
C	2.36103900	-1.17032600	-0.00111800
C	4.13822500	0.97172700	0.00108200
H	2.38032600	2.21675100	0.00183600
C	3.72592600	-1.39553900	-0.00108600
H	1.66263800	-1.99734900	-0.00202200
C	4.62172100	-0.32983100	0.00003200
H	4.82749300	1.80855500	0.00193700
H	4.09964800	-2.41369000	-0.00197800
H	5.68949700	-0.51602900	0.00003700

Azothio-b

C	-4.91481500	-0.44542800	0.01065200
C	-4.54215700	0.86529200	0.01332800
C	-3.13120300	1.03670600	0.00135900
C	-2.46553000	-0.20082800	-0.00743900
S	-3.59744700	-1.52125800	-0.00506100
H	-5.92429700	-0.82980300	0.01629300
H	-5.24362400	1.68946200	0.02234000
N	-2.49621600	2.22146100	-0.01199400
H	-3.00939600	3.08036200	0.07190200
H	-1.48768300	2.20835000	0.03014500
N	-1.14769000	-0.49784500	-0.00805900
N	-0.34559000	0.48304900	-0.01043400
C	1.01235700	0.14095300	-0.00545700
C	1.91740700	1.19807100	-0.00926300
C	1.51151000	-1.16914800	0.00286800
C	3.28706500	0.97907000	-0.00511300
H	1.53190300	2.21177300	-0.01585500
C	2.86852600	-1.39608000	0.00683800
H	0.81873400	-2.00099500	0.00605000
C	3.77128600	-0.32621300	0.00294400
H	3.96061700	1.82527100	-0.00824600
H	3.26350600	-2.40571300	0.01322500
O	5.07923300	-0.65729400	0.00753700
C	6.03018600	0.38962200	0.00403800
H	7.00736900	-0.08891800	0.00865800
H	5.93533400	1.01025100	-0.89227200
H	5.93150500	1.01966500	0.89333600

Z isomers, PBE0-D3/def2tzvp

Azothio-c

C	2.51116900	2.15363500	-0.26658300

C	3.63535600	1.41300700	-0.06865300
C	3.35520800	0.02172500	0.09710600
C	1.97220300	-0.26603200	0.02408300
S	1.03905500	1.24492400	-0.24670100
H	2.46103200	3.22216800	-0.41859700
H	4.63333600	1.82866100	-0.04268400
N	4.29514500	-0.91916100	0.32470800
H	4.00727100	-1.88606400	0.29554600
H	5.26554300	-0.71410200	0.14999300
N	1.57814400	-1.55731400	0.05456900
N	0.41066800	-2.04149500	-0.02506200
C	-0.76183700	-1.23773300	-0.01256700
C	-1.57774600	-1.20410500	-1.15290700
C	-1.20825200	-0.63915500	1.17677000
C	-2.79959300	-0.55101700	-1.12097100
H	-1.24064600	-1.69348000	-2.05845200
C	-2.43630200	0.00195400	1.21792800
H	-0.59332100	-0.69239900	2.06680400
C	-3.23765300	0.05902400	0.06613300
H	-3.42052700	-0.51387000	-2.00725500
H	-2.77954700	0.46036000	2.13695000
C	-4.50032400	0.72387200	0.10516000
N	-5.52272700	1.26352800	0.13606100

Azothio-a

C	2.04337100	1.99872200	-0.05678100
C	3.10624300	1.15133100	-0.01013800
C	2.70667100	-0.20998700	0.02440400
C	1.30895600	-0.35629800	-0.00172900
S	0.52886100	1.22190300	-0.05346700
H	2.08429700	3.07862400	-0.08968100
H	4.13854600	1.47405300	0.00040200
N	3.56446900	-1.24609400	0.11153800
H	3.19072400	-2.17114200	-0.02315300
H	4.53148100	-1.10194000	-0.11998300
N	0.79855900	-1.61065000	-0.02646700
N	-0.40650700	-1.95631100	-0.05122800
C	-1.43934800	-0.97968500	-0.01433900
C	-2.05189200	-0.57441700	-1.19732600
C	-1.92051200	-0.52142300	1.21003600
C	-3.11605400	0.31357200	-1.15397900
H	-1.68250800	-0.95182200	-2.14425400
C	-2.98814000	0.36255900	1.24420500
H	-1.44949200	-0.85731900	2.12707500
C	-3.58488700	0.78808900	0.06439400
H	-3.58263000	0.63510200	-2.07835100
H	-3.35521200	0.72138400	2.19921700
H	-4.41908300	1.47922000	0.09482100

Azothio-b

C	-2.31802300	2.17677100	-0.32690900
C	-3.51322600	1.57174600	-0.09064800

C	-3.37339100	0.17832600	0.13965500
C	-2.03514800	-0.24246600	0.06768600
S	-0.98006600	1.12500400	-0.27116900
H	-2.15922300	3.22673200	-0.53024100
H	-4.46292100	2.08950000	-0.07908600
N	-4.40123800	-0.64636800	0.43218200
H	-4.21407700	-1.63579800	0.41273900
H	-5.33575200	-0.35604300	0.20280300
N	-1.77507700	-1.56821000	0.18266400
N	-0.66262200	-2.14562000	0.13425800
C	0.53392000	-1.38185400	0.01348500
C	1.12369800	-0.80989700	1.13290200
C	1.19345400	-1.30950500	-1.21276500
C	2.33764400	-0.14247800	1.03559400
H	0.62657900	-0.88151400	2.09394600
C	2.39504600	-0.63852500	-1.32014800
H	0.74894700	-1.77367200	-2.08598900
C	2.97564700	-0.04553100	-0.19794300
H	2.77085000	0.29516500	1.92491700
H	2.90702500	-0.56589600	-2.27269500
O	4.15393600	0.58571600	-0.40214200
C	4.77683400	1.20001400	0.70793100
H	5.69355500	1.64930000	0.33113000
H	4.14272600	1.98090000	1.13949500
H	5.02570100	0.46730500	1.48204700

Z-twist isomers, PBE0-D3/def2tzvp

Azothio-c

C	-3.66063900	-1.42180800	0.17028000
C	-2.59601000	-1.77438600	-0.59823300
C	-1.62924600	-0.73957700	-0.73915400
C	-2.00339600	0.40616800	-0.00576200
S	-3.56722800	0.17335400	0.73860400
H	-4.49728000	-2.04890900	0.44205600
H	-2.48670800	-2.74442000	-1.06604900
N	-0.58508000	-0.86991400	-1.58104700
H	0.21114100	-0.25842100	-1.54327500
H	-0.45093600	-1.75153700	-2.04466900
N	-1.55144400	1.68691900	0.09165700
N	-0.36573800	2.08010500	-0.04913100
C	0.75099000	1.23595400	0.00712300
C	1.80421700	1.47514200	-0.88090800
C	0.91091900	0.29055700	1.02800100
C	2.97890400	0.75682700	-0.78260800
H	1.68181200	2.22608800	-1.65212100
C	2.09267900	-0.40828500	1.14531900
H	0.10815200	0.11750800	1.73366000
C	3.13084700	-0.18803900	0.23439400
H	3.78469100	0.93066600	-1.48477500
H	2.21845400	-1.13253800	1.94060300
C	4.34384500	-0.92372300	0.34740000

N	5.32621500	-1.52069100	0.43877800

Azothio-a

C	-3.31883800	-0.95565600	0.27859600
C	-2.30187600	-1.62648900	-0.32537000
C	-1.16689600	-0.81273900	-0.60195200
C	-1.36454200	0.50116100	-0.14822100
S	-2.96681700	0.68747000	0.51494700
H	-4.25971000	-1.36673500	0.61402200
H	-2.34275800	-2.67858700	-0.57798200
N	-0.12446900	-1.28033900	-1.33456700
H	0.78100200	-0.84579500	-1.25137000
H	-0.10495700	-2.27018000	-1.51437200
N	-0.70228500	1.69415900	-0.29991100
N	0.53789300	1.85532300	-0.38507500
C	1.47626000	0.85358200	-0.05190200
C	2.59065900	0.69556700	-0.87623300
C	1.40196200	0.14616500	1.15076500
C	3.59400600	-0.19599700	-0.52674100
H	2.65147500	1.27057000	-1.79331400
C	2.42299500	-0.71929100	1.50438200
H	0.55084400	0.28634400	1.80619400
C	3.51604600	-0.90366200	0.66509200
H	4.44611700	-0.32738100	-1.18358900
H	2.36399100	-1.25727000	2.44369600
H	4.30644200	-1.59032600	0.94393500

Azothio-b

C	3.67857800	-1.52990600	0.00241300
C	2.66479700	-1.65388200	-0.89557700
C	1.75690600	-0.55759500	-0.88395000
C	2.11607300	0.38813500	0.08470000
S	3.59633400	-0.07635300	0.87719800
H	4.46302500	-2.24475300	0.20264700
H	2.55136900	-2.50124200	-1.56015800
N	0.76721000	-0.43871000	-1.81159700
H	-0.05176700	0.10194000	-1.57971000
H	0.58103200	-1.25491300	-2.37073800
N	1.71747600	1.67561100	0.36664800
N	0.55238200	2.13042000	0.27686900
C	-0.59721500	1.32062400	0.14928300
C	-0.80951800	0.17314000	0.91246000
C	-1.64139200	1.79810900	-0.64881500
C	-2.02104800	-0.49670100	0.86605800
H	-0.02628700	-0.19565600	1.56345600
C	-2.83696300	1.11576000	-0.72879200
H	-1.49254200	2.70922700	-1.21721600
C	-3.03964300	-0.03837700	0.03058100
H	-2.15946100	-1.37457600	1.48248900
H	-3.63922500	1.47117800	-1.36443500
O	-4.24218500	-0.63144800	-0.09979000
C	-4.49549900	-1.80167600	0.65484000

H	-5.50594600	-2.11290500	0.39846600
H	-3.79388600	-2.60054800	0.39663800
H	-4.44010700	-1.60154900	1.72906400

PROTONATED SPECIES
E isomers, B3LYP/6-311G(d,p)
Azothio-c

C	-4.57642800	-0.84826300	0.00002400
C	-4.56577600	0.52296700	0.00003400
C	-3.24395400	1.00555200	-0.00000800
C	-2.26773800	0.03191200	-0.00001500
S	-3.00088800	-1.55795100	0.00000400
H	-5.44153400	-1.49322200	0.00003400
H	-5.45217900	1.14050900	0.00004800
N	-0.93146400	0.33292600	-0.00002100
N	-0.14423100	-0.65173400	-0.00001000
C	1.23029900	-0.34070600	-0.00001500
C	2.08913700	-1.44768400	-0.00001800
C	1.75866000	0.96144900	0.00000800
C	3.46391300	-1.26808200	-0.00001200
H	1.65849000	-2.44078400	-0.00002400
C	3.12868900	1.14622900	0.00001600
H	1.09263100	1.81269100	0.00002100
C	3.98847100	0.03207200	0.00000200
H	4.12878100	-2.12168400	-0.00001700
H	3.54481200	2.14539400	0.00003400
C	5.40413800	0.22938800	0.00001100
N	6.54845400	0.39043200	0.00001800
H	-3.20730900	2.91544300	0.82719900
H	-1.83495100	2.47998900	-0.00005100
H	-3.20735000	2.91543500	-0.82722800
N	-2.86185200	2.41758100	-0.00002000

Azothio-a

C	3.86102500	-0.96214600	-0.00013000
C	3.90712000	0.40651600	0.00011900
C	2.60455500	0.94532700	0.00011800
C	1.58832500	0.01672000	-0.00002900
S	2.25287600	-1.60301200	-0.00024400
H	4.69687500	-1.64449500	-0.00024300
H	4.81812300	0.98745200	0.00023300
N	0.26327100	0.37533900	0.00005600
N	-0.56171700	-0.57827200	0.00004500
C	-1.92413200	-0.22707200	-0.00001200
C	-2.81727400	-1.30777700	0.00036300
C	-2.41494000	1.09052000	-0.00040800
C	-4.18901200	-1.07940900	0.00037900
H	-2.41341000	-2.31288600	0.00064800
C	-3.78396000	1.30867700	-0.00041300
H	-1.72100500	1.92009200	-0.00073100
C	-4.67362100	0.22772000	-0.00001200

H	-4.87692800	-1.91621100	0.00068600
H	-4.16698000	2.32237600	-0.00073600
H	-5.74198500	0.40936400	-0.00001400
H	1.25506300	2.47172300	0.00023100
H	2.64388500	2.85614500	0.82730400
H	2.64410300	2.85644200	-0.82635400
N	2.27955200	2.37203700	0.00033400

Azothio-b

C	4.71212400	-0.99802100	0.00018800
C	4.77387100	0.36869700	-0.00018300
C	3.47654500	0.92426400	-0.00024500
C	2.44727800	0.01114800	0.00001000
S	3.09231100	-1.61786700	0.00040400
H	5.53837300	-1.69180400	0.00048900
H	5.69156500	0.93924100	-0.00027500
N	1.12808600	0.38815300	-0.00006000
N	0.28934600	-0.55994300	-0.00002700
C	-1.05887200	-0.20623500	0.00011300
C	-1.96711300	-1.27450000	-0.00054500
C	-1.55451500	1.11642100	0.00086000
C	-3.33708500	-1.05397900	-0.00065500
H	-1.57512300	-2.28434800	-0.00107300
C	-2.91138400	1.34357200	0.00082500
H	-0.86109600	1.94648100	0.00148700
C	-3.81941500	0.26241300	0.00002600
H	-4.01376600	-1.89646000	-0.00125900
H	-3.31247100	2.34968500	0.00143000
O	-5.12328100	0.60097800	-0.00007500
C	-6.11369900	-0.43622300	-0.00013100
H	-7.07226000	0.07685200	0.00044600
H	-6.02949700	-1.05705000	-0.89560500
H	-6.02880500	-1.05781000	0.89474600
H	3.53291600	2.83585500	-0.82742700
H	2.14099500	2.45867700	-0.00110700
H	3.53233000	2.83627500	0.82608300
N	3.16538500	2.35423200	-0.00068400

E-twist isomers, B3LYP/6-311G(d,p)

Azothio-c

C	4.73904400	-0.57451600	0.02106700
C	4.39258800	0.75585100	-0.00243500
C	2.99792400	0.91798500	-0.01312800
C	2.28226500	-0.26840900	0.00231800
S	3.37814500	-1.62118800	0.03013400
H	5.73349700	-0.99229300	0.03323000
H	5.10599800	1.56790500	-0.01209100
N	0.93165200	-0.54327000	0.00110100
N	0.17718600	0.47264300	-0.00725400
C	-1.20797800	0.21267200	-0.00859300
C	-2.03915000	1.33874900	0.04097500
C	-1.76327400	-1.07675500	-0.05746500

C	-3.41747800	1.18813700	0.04892300
H	-1.59319500	2.32451000	0.07648800
C	-3.13692500	-1.23078200	-0.05262600
H	-1.11151500	-1.93798900	-0.09973600
C	-3.97134100	-0.09881900	0.00217100
H	-4.06282200	2.05553600	0.09060300
H	-3.57488000	-2.21976800	-0.09136600
C	-5.39114800	-0.26359700	0.00933700
N	-6.53884100	-0.39802000	0.01568000
H	1.28668900	2.00568700	-0.03928900
H	2.54099300	2.79265700	0.76506900
H	2.53996400	2.74785700	-0.88922700
N	2.31254500	2.21033500	-0.04653800

Azothio-a

C	-4.08912100	-0.43608700	0.00004700
C	-3.67682200	0.87344200	-0.00006300
C	-2.27303600	0.96563400	-0.00007200
C	-1.61646200	-0.25205900	0.00001600
S	-2.77724400	-1.54914500	0.00012400
H	-5.10231100	-0.80598700	0.00009000
H	-4.34796800	1.72108800	-0.00012400
N	-0.27607200	-0.59215100	0.00001400
N	0.52038800	0.39136500	0.00001300
C	1.89506100	0.09046700	0.00000600
C	2.76025800	1.19312900	0.00018900
C	2.41431000	-1.21558600	-0.00018900
C	4.13678500	0.99561800	0.00019600
H	2.34050000	2.19182700	0.00033000
C	3.78797100	-1.40109200	-0.00018900
H	1.73612600	-2.05794000	-0.00034600
C	4.65203500	-0.29964700	0.00000700
H	4.80458700	1.84845700	0.00034500
H	4.19430000	-2.40555100	-0.00034700
H	5.72433600	-0.45581900	0.00000500
H	-1.71429900	2.79160900	0.82696900
H	-0.50551900	1.95149200	0.00004600
H	-1.71405000	2.79128600	-0.82759800
N	-1.51941000	2.21977900	-0.00017500

Azothio-b

C	4.95380500	-0.39747900	0.00007600
C	4.52350600	0.90425300	0.00000100
C	3.11622100	0.97854700	-0.00001100
C	2.47369900	-0.24577000	-0.00002000
S	3.65251000	-1.52816400	0.00006500
H	5.97124000	-0.75523000	0.00013100
H	5.18293800	1.76114600	0.00001100
N	1.13816600	-0.60459600	-0.00004400
N	0.32937100	0.37537100	-0.00004400
C	-1.03195300	0.07447300	-0.00005100
C	-1.91049500	1.16680500	-0.00001200

C	-1.55871000	-1.23555400	-0.00009200
C	-3.28533400	0.97980300	-0.00000200
H	-1.50064700	2.16966200	0.00001500
C	-2.92066200	-1.42782200	-0.00008400
H	-0.88269700	-2.07965000	-0.00012900
C	-3.80116100	-0.32398200	-0.00003100
H	-3.94018500	1.83932000	0.00001400
H	-3.34671800	-2.42351400	-0.00011500
O	-5.11256500	-0.62894900	-0.00002100
C	-6.07694700	0.43289900	0.00016200
H	-7.04787100	-0.05625000	0.00028400
H	-5.97655200	1.05143800	0.89546600
H	-5.97680900	1.05151100	-0.89512100
H	2.53039600	2.79753200	-0.82721900
H	1.33530000	1.93590100	-0.00003300
H	2.53033500	2.79754000	0.82714300
N	2.34529400	2.22290600	-0.00004400

Z isomers, B3LYP/6-311G(d,p)

Azothio-c

C	2.34633800	2.24174200	-0.02150600
C	3.51266000	1.51793400	-0.00467200
C	3.23513900	0.14346900	0.00637600
C	1.89387400	-0.19721300	-0.00033100
S	0.92610200	1.26963600	-0.02197100
H	2.24499200	3.31645500	-0.03349100
H	4.50075400	1.95419000	-0.00131600
N	1.57542500	-1.54727000	0.00169100
N	0.43952000	-2.06404400	-0.00266400
C	-0.75761100	-1.27814700	-0.00052100
C	-1.39116000	-0.99120300	-1.21432400
C	-1.36033900	-0.94574400	1.21752800
C	-2.61269000	-0.33383800	-1.21080900
H	-0.92677200	-1.28190200	-2.14813700
C	-2.58268300	-0.28982400	1.22027800
H	-0.87305200	-1.20145300	2.14993500
C	-3.21196800	0.02428700	0.00626500
H	-3.10441400	-0.09966400	-2.14616400
H	-3.05146900	-0.02202900	2.15838400
C	-4.47101500	0.70117400	0.00956200
N	-5.48797100	1.25007300	0.01210500
H	4.82349100	-0.88650800	0.86541500
H	3.73755000	-1.81975200	0.00807500
H	4.86333200	-0.87790500	-0.78926700
N	4.23941100	-0.91753400	0.02329900

Azothio-a

C	2.14242700	2.04062100	-0.17127800
C	3.17089100	1.13926400	-0.06054500
C	2.66675100	-0.16741500	0.03138200
C	1.28877500	-0.28422900	0.00259400
S	0.57920000	1.31509100	-0.15051100

H	2.22094300	3.11375800	-0.25940100
H	4.21823200	1.40372600	-0.04871100
N	0.76125600	-1.57151300	0.01293000
N	-0.44018200	-1.90882200	-0.02381000
C	-1.49786800	-0.93918000	0.01129800
C	-2.24633900	-0.71454400	-1.14751600
C	-1.86841000	-0.35124400	1.22527200
C	-3.34052600	0.14491900	-1.09735900
H	-1.96396800	-1.20503000	-2.07129900
C	-2.97741200	0.49018200	1.26473400
H	-1.30163200	-0.56250200	2.12436100
C	-3.70868700	0.74774600	0.10568500
H	-3.91193000	0.33440900	-1.99846000
H	-3.26909600	0.94362100	2.20481600
H	-4.56899100	1.40535000	0.14195400
H	4.01573400	-1.42219500	1.00263000
H	2.81844600	-2.17963000	0.11834100
H	4.12987300	-1.48059000	-0.64820100
N	3.47429500	-1.38096400	0.13324400

Azothio-b

C	-2.64519800	2.14818000	-0.57422600
C	-3.73979400	1.44144100	-0.15114700
C	-3.37245900	0.12861200	0.19125800
C	-2.03068400	-0.18111700	0.05968300
S	-1.17720300	1.23854000	-0.53126000
H	-2.61579300	3.17464900	-0.90693900
H	-4.74210000	1.84102200	-0.09699200
N	-1.67912900	-1.51992200	0.21741700
N	-0.54956300	-2.05117700	0.11063700
C	0.65349200	-1.30278900	0.01585300
C	1.02847400	-0.36115300	0.98011400
C	1.57428900	-1.67456200	-0.97799400
C	2.28261100	0.23893800	0.93182800
H	0.35828200	-0.12222600	1.79676100
C	2.80370100	-1.04951700	-1.05621500
H	1.30025200	-2.44222200	-1.69150800
C	3.17107200	-0.08479800	-0.10199800
H	2.55685200	0.94528200	1.70272900
H	3.51116200	-1.30369100	-1.83578800
O	4.40345000	0.45309500	-0.25351600
C	4.84718200	1.43977100	0.68436500
H	5.84434700	1.72596800	0.35795600
H	4.19275800	2.31566900	0.67291400
H	4.89811800	1.02753300	1.69581100
H	-3.74360100	-1.80147400	0.63973100
H	-4.67016400	-0.75939200	1.55914700
H	-5.08200500	-1.04116600	-0.01899600
N	-4.29081800	-0.92309900	0.62113500

Z-twist isomers, B3LYP/6-311G(d,p)

Azothio-c

C	3.49113300	-1.55252700	-0.20175000
C	2.60768400	-1.66815300	0.83341300
C	1.76342200	-0.53724400	0.93008900
C	1.99655200	0.43156500	-0.02626900
S	3.33937700	-0.04872800	-1.02866800
H	4.22163300	-2.27326400	-0.53382300
H	2.55976600	-2.52100900	1.49667300
N	1.55678500	1.76068000	-0.19489800
N	0.36343500	2.12357800	-0.15810700
C	-0.74687400	1.22655700	-0.14716200
C	-1.80174400	1.53259800	0.72473700
C	-0.88439100	0.20022100	-1.09547000
C	-2.96717700	0.77970500	0.69374100
H	-1.69657900	2.36063200	1.41443200
C	-2.06165300	-0.52938000	-1.14754100
H	-0.09258600	-0.00341100	-1.80386000
C	-3.10191000	-0.25254700	-0.24596300
H	-3.77542000	1.00094800	1.37819800
H	-2.17985900	-1.31264200	-1.88479000
C	-4.31026900	-1.01528500	-0.29725200
N	-5.28613500	-1.63221000	-0.33692400
H	1.10029200	0.27706600	2.72997100
H	-0.13423300	-0.10027800	1.69534600
H	0.66408600	-1.29495500	2.50793500
N	0.79366400	-0.40154900	2.02352400

Azothio-a

C	3.21999500	0.93387800	0.54009600
C	2.33914000	1.60849800	-0.25461300
C	1.28755000	0.77031100	-0.69805200
C	1.35702800	-0.52787600	-0.23842600
S	2.80111500	-0.73182600	0.71501900
H	4.09191500	1.32069100	1.04332200
H	2.43189100	2.65456500	-0.51331700
N	0.66077000	-1.70806600	-0.58412900
N	-0.58443800	-1.80804200	-0.61509700
C	-1.47696300	-0.81451300	-0.10516900
C	-2.63637600	-0.56742200	-0.85515600
C	-1.32038300	-0.23696800	1.16543000
C	-3.60184500	0.30845900	-0.36634100
H	-2.76149300	-1.06428600	-1.80971700
C	-2.31167700	0.60446500	1.65728700
H	-0.45485600	-0.47122800	1.77115200
C	-3.44332600	0.89090200	0.89080100
H	-4.48551900	0.51629900	-0.95714300
H	-2.20166400	1.03582500	2.64488600
H	-4.20623400	1.55363600	1.28089500
H	0.36459100	0.87422800	-2.56691700
H	-0.69513500	1.01489100	-1.30322500
H	0.29674400	2.27566900	-1.70534700
N	0.25553400	1.25618200	-1.62077900

Azothio-b

C	-3.57939800	-1.61784500	-0.00713400
C	-2.76250400	-1.50160200	1.07895100
C	-1.94996900	-0.34367000	1.00099000
C	-2.13178300	0.41254500	-0.13735600
S	-3.39208000	-0.29931600	-1.10956100
H	-4.27955700	-2.40674600	-0.23139500
H	-2.73759900	-2.20787600	1.89764200
N	-1.70708700	1.70909200	-0.49538500
N	-0.51849200	2.10884800	-0.45584400
C	0.61649900	1.27911600	-0.27221200
C	0.77022000	-0.00642000	-0.82071400
C	1.72631100	1.89547100	0.34106400
C	1.98316600	-0.67406100	-0.72180500
H	-0.03915200	-0.47438500	-1.36454900
C	2.92083300	1.21885000	0.47855200
H	1.61852300	2.90762200	0.71127800
C	3.06389500	-0.07521600	-0.05348400
H	2.08225900	-1.65002400	-1.17528700
H	3.77037600	1.67552700	0.97027800
O	4.27218100	-0.64677800	0.10915500
C	4.50293800	-1.96007700	-0.42031000
H	5.53081700	-2.20081000	-0.16059500
H	3.82835700	-2.68933100	0.03511500
H	4.38703700	-1.96976700	-1.50692900
H	-0.06429700	0.17891800	1.71265000
H	-1.30136500	0.87521800	2.56764900
H	-0.96184600	-0.72006800	2.77557600
N	-1.01713100	0.02241100	2.07252200

E isomers, PBE0-D3/def2tzvp

Azothio-c

C	-4.54854300	-0.83307700	-0.01327600
C	-4.53400900	0.53533100	0.01939300
C	-3.21627000	1.00412400	0.02439100
C	-2.25587000	0.02109300	-0.00316100
S	-2.99689800	-1.53451300	-0.03636600
H	-5.41999800	-1.47076100	-0.02521100
H	-5.41733400	1.15754900	0.03799300
N	-0.92240800	0.31497800	0.00016600
N	-0.14578700	-0.66284100	-0.00941200
C	1.22170300	-0.35101500	-0.01016900
C	2.07843600	-1.44851500	0.05496300
C	1.73914800	0.94641500	-0.07580000
C	3.44681800	-1.26339900	0.06363600
H	1.65157600	-2.44273700	0.10133200
C	3.10267800	1.13644400	-0.07108800
H	1.06951200	1.79378200	-0.13335300
C	3.96002300	0.03191200	0.00051000
H	4.11703300	-2.11150300	0.11753400
H	3.51661100	2.13549400	-0.12339100

C	5.37103200	0.23548100	0.00642300
N	6.51129600	0.40150900	0.01155800
H	-3.15573000	2.87509200	0.89963800
H	-1.79462200	2.44599700	0.05455500
H	-3.16580300	2.91878600	-0.75113700
N	-2.82049500	2.39803100	0.05896700

Azothio-a

C	3.83321800	-0.95659100	-0.00006700
C	3.88226700	0.40959800	-0.00002000
C	2.58602900	0.94126900	0.00002300
C	1.58036600	0.00722900	0.00002900
S	2.24649200	-1.58217700	-0.00003900
H	4.67255600	-1.63597900	-0.00010300
H	4.79337100	0.99095700	-0.00002500
N	0.25875000	0.36293900	0.00004400
N	-0.55678600	-0.58296400	0.00000800
C	-1.91210500	-0.23091900	0.00000900
C	-2.80273100	-1.30389000	0.00008700
C	-2.39337700	1.08254300	-0.00008700
C	-4.16820900	-1.07179700	0.00007600
H	-2.40234500	-2.31096900	0.00015900
C	-3.75605700	1.30433700	-0.00010700
H	-1.69679600	1.91077600	-0.00015600
C	-4.64565300	0.23131000	-0.00002500
H	-4.85925600	-1.90615500	0.00014100
H	-4.13563600	2.31945700	-0.00019100
H	-5.71345300	0.41699700	-0.00004800
H	1.23275800	2.44606200	0.00016700
H	2.61860100	2.83639100	0.82540500
H	2.61847700	2.83646200	-0.82523500
N	2.25594300	2.35321100	0.00009200

Azothio-b

C	4.65711000	-1.02235300	0.00433200
C	4.74062100	0.34095800	0.01270200
C	3.46024700	0.91208300	0.00652500
C	2.42589100	0.01022700	-0.00504000
S	3.05108700	-1.59829900	-0.00811900
H	5.47719700	-1.72452200	0.00574100
H	5.66788100	0.89729000	0.02226300
N	1.11058100	0.38752600	-0.00769300
N	0.28704500	-0.55845000	-0.00783700
C	-1.05584300	-0.20687100	-0.00844500
C	-1.96099500	-1.26693900	0.00486000
C	-1.54141800	1.11133000	-0.02011500
C	-3.32517700	-1.04268700	0.00877000
H	-1.57277400	-2.27889500	0.01337100
C	-2.89221500	1.34259200	-0.01748500
H	-0.84276400	1.93811500	-0.03161400
C	-3.79922100	0.26955500	-0.00217800
H	-4.00560100	-1.88252600	0.02017800

H	-3.28802000	2.35119400	-0.02689100
O	-5.09440800	0.60647300	0.00056800
C	-6.06506200	-0.42805800	0.01801000
H	-7.03214700	0.06921800	0.01846300
H	-5.98105300	-1.06131100	-0.86928800
H	-5.96995400	-1.04134500	0.91812400
H	2.68699900	2.63790100	-0.81737800
H	2.64574800	2.61944800	0.82556800
H	4.07630100	2.87303900	0.04279500
N	3.20414900	2.34297600	0.01444500

E-twist isomers, PBE0-D3/def2tzvp

Azothio-c

C	4.70546100	-0.57369100	-0.03846900
C	4.36363600	0.75492600	0.01256100
C	2.97756400	0.91345500	0.03038300
C	2.27187900	-0.27419900	-0.00651200
S	3.35992500	-1.59853200	-0.06280100
H	5.70121700	-0.98956500	-0.06202400
H	5.07808500	1.56631900	0.03604700
N	2.28632700	2.18600900	0.09423800
H	2.50894900	2.78899900	-0.70040000
H	1.26367700	1.96010500	0.07888500
N	0.92518100	-0.54411800	-0.00810200
N	0.18174600	0.46509400	0.01184500
C	-1.19726300	0.21039600	0.01372300
C	-2.02098700	1.32968200	-0.08719900
C	-1.74866600	-1.06962600	0.11437200
C	-3.39361300	1.18041200	-0.10115400
H	-1.57315200	2.31299100	-0.16089100
C	-3.11688700	-1.22206700	0.10651800
H	-1.09856800	-1.92965300	0.20160800
C	-3.94331600	-0.09759800	-0.00417600
H	-4.03957700	2.04460900	-0.18537100
H	-3.55794000	-2.20746800	0.18682900
C	-5.35967600	-0.26143000	-0.01514400
N	-6.50419900	-0.39473900	-0.02483800
H	2.50632400	2.70290600	0.94871900

Azothio-a

C	-4.06056300	-0.42907900	0.00019700
C	-3.64738100	0.87829400	-0.00018300
C	-2.25213100	0.96188900	-0.00027100
C	-1.61121000	-0.26044600	0.00007700
S	-2.76780300	-1.52555500	0.00046100
H	-5.07709600	-0.79202500	0.00038500
H	-4.31588700	1.72837300	-0.00050100
N	-0.27568600	-0.59929600	0.00024300
N	0.51061500	0.37672400	-0.00003700
C	1.87882000	0.08339000	-0.00004600
C	2.73242900	1.18550900	0.00069500
C	2.39893700	-1.21438900	-0.00082400

C	4.10429800	0.99602100	0.00082600
H	2.30597200	2.18188400	0.00123500
C	3.76793700	-1.39206600	-0.00075200
H	1.72549300	-2.06152900	-0.00152600
C	4.62282300	-0.29127000	0.00010900
H	4.76810500	1.85207600	0.00146400
H	4.17940000	-2.39455600	-0.00139800
H	5.69595300	-0.44257300	0.00017400
H	-1.67468800	2.76613400	0.82480300
H	-0.47909200	1.89886500	-0.00039300
H	-1.67421300	2.76517900	-0.82702800
N	-1.48819300	2.19399000	-0.00071400

Azothio-b

C	-4.95222100	-0.44789200	0.01969100
C	-4.57607200	0.86311400	0.02322300
C	-3.15661000	1.04107600	0.00191000
C	-2.47706800	-0.19408600	-0.01287400
S	-3.61825500	-1.54610500	-0.00777600
H	-5.95945800	-0.83606300	0.02979100
H	-5.27772900	1.68689700	0.03784000
N	-1.15579900	-0.48983200	-0.01369800
N	-0.34440900	0.50138500	-0.01815800
C	1.02033000	0.15387600	-0.00947000
C	1.93501500	1.21244600	-0.01445300
C	1.52038700	-1.16359100	0.00291500
C	3.30970700	0.98714600	-0.00742200
H	1.55439500	2.22745800	-0.02436000
C	2.88209700	-1.39647100	0.00957300
H	0.82356600	-1.99095100	0.00688800
C	3.79291000	-0.32507100	0.00465600
H	3.98540300	1.83127400	-0.01151800
H	3.27520400	-2.40650500	0.01894200
O	5.10923300	-0.66772000	0.01230700
C	6.08606500	0.37507100	0.00864900
H	7.05225600	-0.12510000	0.01641000
H	6.00311900	0.99316900	-0.89030200
H	5.99560200	1.00641900	0.89761000
H	-1.88259926	2.37406584	0.84364696
H	-1.89873334	2.34747720	-0.90337924
H	-3.22923176	3.06649067	-0.02824486
N	-2.50316204	2.28055483	-0.02298879

Z isomers, PBE0-D3/def2tzvp

Azothio-c

C	2.24180100	2.23315700	0.00054600
C	3.42987100	1.55092000	-0.00033400
C	3.19290600	0.17697200	-0.00035200
C	1.86397900	-0.19368000	0.00032600
S	0.87678000	1.22587500	0.00112200
H	2.10851600	3.30510200	0.00089400
H	4.40444700	2.01754900	-0.00083600

N	4.21228300	-0.85172600	-0.00127400
H	3.72872200	-1.76195000	0.00030800
H	4.81192200	-0.80252100	-0.82847300
N	1.58303600	-1.54572700	0.00046500
N	0.46200600	-2.07198400	0.00050600
C	-0.72716300	-1.29735300	0.00025500
C	-1.33792200	-0.98305900	-1.21117100
C	-1.33888900	-0.98370900	1.21134900
C	-2.54858500	-0.31858200	-1.21108800
H	-0.86223300	-1.25797900	-2.14445100
C	-2.54954600	-0.31921700	1.21063000
H	-0.86394600	-1.25912500	2.14486300
C	-3.15540800	0.01941600	-0.00038300
H	-3.02759900	-0.06207600	-2.14745000
H	-3.02931400	-0.06319900	2.14673800
C	-4.40559200	0.70476700	-0.00072100
N	-5.41544600	1.25994700	-0.00074900
H	4.81513700	-0.80114700	0.82349700

Azothio-a

C	1.93343000	2.06340100	-0.00016600
C	3.02355200	1.23279800	-0.00026700
C	2.61432400	-0.10028300	-0.00008500
C	1.24994600	-0.30123900	0.00001900
S	0.45397100	1.23638800	0.00011800
H	1.94136100	3.14367300	-0.00018900
H	4.05011500	1.57129700	-0.00054300
N	0.79057600	-1.60844600	-0.00004900
N	-0.39664000	-1.96668300	-0.00031700
C	-1.44577300	-1.00362300	-0.00014300
C	-1.98456600	-0.57732800	-1.20974800
C	-1.98425700	-0.57740400	1.20963300
C	-3.04550500	0.31509000	-1.20181200
H	-1.56549700	-0.93687400	-2.14229300
C	-3.04518700	0.31502700	1.20204600
H	-1.56494700	-0.93703700	2.14203600
C	-3.57334300	0.76744300	0.00019900
H	-3.46142300	0.65715500	-2.14231000
H	-3.46087300	0.65700800	2.14267600
H	-4.40278800	1.46453100	0.00032600
H	3.38978900	-1.82164300	0.82304400
H	3.38514500	-1.82589900	-0.81865000
H	4.50354900	-0.92227600	-0.00319700
N	3.52982100	-1.22993400	0.00024700

Azothio-b

C	-2.36317300	2.17940100	-0.51782500
C	-3.53253900	1.55831300	-0.17224300
C	-3.28725100	0.22113500	0.14703700
C	-1.97082000	-0.17998700	0.06398400
S	-1.00159600	1.16379800	-0.43093800
H	-2.23864200	3.21054700	-0.81495800

H	-4.50179500	2.03570900	-0.15557500
N	-1.71992300	-1.53376600	0.22913900
N	-0.62184700	-2.10647400	0.14692900
C	0.58189400	-1.36712200	0.03485900
C	1.02464900	-0.56102400	1.07683600
C	1.39819000	-1.57064600	-1.07771100
C	2.25400200	0.07416500	0.99952400
H	0.41250100	-0.43867000	1.96305100
C	2.60490900	-0.91304800	-1.17453100
H	1.06666500	-2.22954000	-1.87155800
C	3.04393900	-0.08434200	-0.13782400
H	2.58324300	0.68558200	1.82826200
H	3.23704000	-1.03842200	-2.04536800
O	4.24137200	0.50159200	-0.31861500
C	4.73190300	1.35124500	0.70238500
H	5.69290400	1.72024800	0.35074900
H	4.05855100	2.19672800	0.87178000
H	4.87460500	0.80453600	1.63919900
H	-3.80839700	-1.66738800	0.56820800
H	-4.72303100	-0.55924300	1.41678000
H	-5.03954700	-0.82214400	-0.18273700
N	-4.29162500	-0.75690300	0.51095600

Z-twist isomers, PBE0-D3/def2tzvp

Azothio-c

C	3.40196900	1.58006000	-0.18385300
C	2.51475600	1.66958400	0.84746900
C	1.70763900	0.52207300	0.93450000
C	1.97825100	-0.42843200	-0.02544200
S	3.28906600	0.09740600	-1.00151800
H	4.11441600	2.32343800	-0.50711000
H	2.44481800	2.51753200	1.51556300
N	0.74475000	0.35272600	2.01090400
H	-0.17365200	0.03836600	1.67072200
H	1.05920800	-0.33158500	2.70500400
N	1.57012700	-1.75822200	-0.20760000
N	0.39030200	-2.13180700	-0.18480700
C	-0.71502300	-1.24595500	-0.17071700
C	-0.85297800	-0.22991000	-1.11947200
C	-1.75468500	-1.53540100	0.71416600
C	-2.01879400	0.50736800	-1.16179600
H	-0.06544000	-0.03806000	-1.83682500
C	-2.90829100	-0.77406000	0.69304900
H	-1.64810900	-2.35753600	1.41139600
C	-3.04299700	0.24677600	-0.24808200
H	-2.14020800	1.28802700	-1.90172100
H	-3.71026200	-0.98101800	1.38969200
C	-4.24080400	1.01998600	-0.28713600
N	-5.20778400	1.64529800	-0.31660000
H	0.59814900	1.23384100	2.50817400

Azothio-a

C	3.12566200	1.01664500	0.44599700
C	2.20480000	1.61620300	-0.36079700
C	1.19197400	0.71807400	-0.74213100
C	1.33847000	-0.54832300	-0.22291500
S	2.78376900	-0.62886300	0.69777300
H	3.98926800	1.46750500	0.91040400
H	2.24893700	2.65279000	-0.66730700
N	0.68099000	-1.76746500	-0.46218000
N	-0.55155600	-1.89844100	-0.45165500
C	-1.43258000	-0.87447900	-0.01731100
C	-2.56809900	-0.63966900	-0.79326900
C	-1.25778500	-0.21142900	1.19933300
C	-3.49353200	0.30908700	-0.38341600
H	-2.70386100	-1.19360100	-1.71482300
C	-2.20874100	0.70492100	1.61332900
H	-0.39922600	-0.43027400	1.82179800
C	-3.31693200	0.97885300	0.81941300
H	-4.36150600	0.51115400	-0.99905100
H	-2.08289600	1.20970800	2.56357900
H	-4.04982000	1.70602400	1.14727600
H	0.20819800	0.65516800	-2.56229500
H	-0.80409600	0.85956900	-1.27294100
H	0.13484900	2.11554200	-1.81093600
N	0.12491000	1.10718200	-1.64751800

Azothio-b

C	-3.38584100	-1.69296400	0.06725300
C	-2.51572600	-1.54224800	1.10509700
C	-1.78487500	-0.34535500	0.99315700
C	-2.09109100	0.40353800	-0.11961200
S	-3.33974800	-0.37213700	-1.00486200
H	-4.05063400	-2.52147900	-0.12297600
H	-2.40173700	-2.25278300	1.91301300
N	-1.75162200	1.71249000	-0.50079900
N	-0.58885000	2.15143600	-0.50506800
C	0.55183600	1.33740500	-0.33366900
C	0.72188400	0.10441200	-0.96753400
C	1.61360000	1.88380500	0.39804600
C	1.90958200	-0.59011700	-0.83935600
H	-0.06339700	-0.30237200	-1.59207200
C	2.78195300	1.17485100	0.56449100
H	1.49299500	2.86138000	0.84998100
C	2.94224600	-0.06983900	-0.05295400
H	2.02607700	-1.53077700	-1.35932400
H	3.59708300	1.57491300	1.15456400
O	4.11603400	-0.67896900	0.14872400
C	4.33900800	-1.94240100	-0.45752400
H	5.33671800	-2.24785200	-0.15146600
H	3.60963600	-2.67931200	-0.11062100
H	4.29952400	-1.86849900	-1.54755000
H	0.05873900	0.38605200	1.56286500

H	-1.16820400	0.83501600	2.57817900
H	-0.59435700	-0.70736000	2.62474100
N	-0.82098600	0.06643000	1.99790900

E isomers, B3LYP/def2svp

Azothio-a

C	3.86573100	-0.96772000	0.00008700
C	3.91652900	0.40794000	0.00005300
C	2.61209500	0.95255700	-0.00003800
C	1.59143800	0.01704400	-0.00004600
S	2.25749000	-1.60195900	0.00006700
H	4.71162600	-1.65437000	0.00011500
H	4.83746000	0.99050000	0.00009400
N	0.26697500	0.37131900	-0.00002000
N	-0.56328700	-0.57579100	0.00002000
C	-1.92399300	-0.22896200	0.00000000
C	-2.82191900	-1.31312900	-0.00016100
C	-2.41756100	1.09345900	0.00017600
C	-4.19797100	-1.08376300	-0.00016300
H	-2.41224400	-2.32524300	-0.00029300
C	-3.79153200	1.31246000	0.00019000
H	-1.71698400	1.92888200	0.00030100
C	-4.68415800	0.22816200	0.00002300
H	-4.89222400	-1.92703700	-0.00030200
H	-4.17789300	2.33452800	0.00033900
H	-5.76136100	0.41131600	0.00005600
H	1.24711100	2.46078600	-0.00002200
H	2.63850400	2.86759300	0.82892900
H	2.63819000	2.86729200	-0.82964000
N	2.27860100	2.37144400	-0.00019700

Azothio-b

C	4.71923800	-1.00222200	-0.00012100
C	4.78502100	0.37162300	-0.00014800
C	3.48567600	0.93209600	-0.00002200
C	2.45249700	0.01119500	0.00006600
S	3.10004200	-1.61637600	0.00002400
H	5.55601900	-1.69977600	-0.00018100
H	5.71234900	0.94413600	-0.00024100
N	1.13376800	0.38342500	0.00014300
N	0.29013800	-0.55802400	0.00007700
C	-1.05711400	-0.20847500	0.00009400
C	-1.97033700	-1.28019000	0.00004100
C	-1.55595900	1.11853900	0.00009100
C	-3.34448200	-1.05921500	-0.00000300
H	-1.57223300	-2.29701800	0.00004100
C	-2.91810100	1.34613100	0.00003400
H	-0.85622300	1.95478000	0.00014500
C	-3.83101800	0.26221800	-0.00003100
H	-4.02575400	-1.90923800	-0.00000900
H	-3.32299800	2.36022100	0.00003200
O	-5.13154800	0.59305700	-0.00015100

C	-6.12383700	-0.42583700	-0.00006000
H	-7.09234000	0.08900000	-0.00005400
H	-6.04821600	-1.05871200	-0.89944800
H	-6.04814800	-1.05861200	0.89939200
H	3.52562000	2.84823700	-0.82927400
H	2.13233100	2.44575700	0.00007300
H	3.52580300	2.84826000	0.82917200
N	3.16409400	2.35393500	-0.00000200

Azothio-c

C	-4.58388500	-0.85444600	-0.00000100
C	-4.57812500	0.52380300	-0.00006500
C	-3.25419400	1.01285500	-0.00008900
C	-2.27342600	0.03279600	-0.00000400
S	-3.00730000	-1.55674600	0.00006600
H	-5.45879900	-1.50382300	0.00000400
H	-5.47461600	1.14308900	-0.00009000
N	-0.93761700	0.32968300	0.00000300
N	-0.14482200	-0.64859200	0.00015300
C	1.22765800	-0.34251000	0.00011500
C	2.09128300	-1.45294300	-0.00010900
C	1.75981500	0.96399000	0.00031000
C	3.47060500	-1.27290700	-0.00018500
H	1.65449800	-2.45290500	-0.00022400
C	3.13494600	1.14903500	0.00025400
H	1.08762000	1.82162000	0.00052300
C	3.99942700	0.03177400	-0.00000400
H	4.14011000	-2.13423800	-0.00037800
H	3.55350200	2.15685500	0.00041900
C	5.42110600	0.22973700	-0.00008900
N	6.57146500	0.39148800	-0.00016100
H	-3.20636800	2.92605900	0.82937700
H	-1.83173000	2.47067700	-0.00011700
H	-3.20622200	2.92593300	-0.82991800
N	-2.86509000	2.41707300	-0.00020000

E-twist isomers, B3LYP/def2svp

Azothio-a

C	-4.09872300	-0.41854700	0.00022600
C	-3.67404600	0.89460300	0.00000900
C	-2.26567600	0.97428900	-0.00013600
C	-1.62223300	-0.26038900	-0.00003700
S	-2.79895600	-1.54313700	0.00023800
H	-5.12678900	-0.77811200	0.00038300
H	-4.34425400	1.75457700	-0.00002800
N	-0.28428900	-0.59474300	-0.00004600
N	0.51707200	0.38309500	0.00005900
C	1.89081900	0.09046500	0.00005300
C	2.76152900	1.19598400	0.00050400
C	2.41116700	-1.22087400	-0.00041900
C	4.14214900	0.99519500	0.00050100
H	2.34023000	2.20341800	0.00087100

C	3.78935100	-1.40921400	-0.00044500
H	1.72386000	-2.06717000	-0.00079400
C	4.65762900	-0.30539400	0.00002000
H	4.81697100	1.85396200	0.00086700
H	4.19786600	-2.42251000	-0.00084100
H	5.73861300	-0.46404100	-0.00000400
H	-1.65456200	2.78735300	0.82897300
H	-0.46619100	1.88520100	-0.00008300
H	-1.65398800	2.78629600	-0.83097200
N	-1.48139200	2.20229200	-0.00056100

Azothio-b

C	4.96390900	-0.38279000	0.00028700
C	4.52237100	0.92288700	-0.00021900
C	3.11087900	0.98589400	-0.00024800
C	2.48051500	-0.25466200	0.00003500
S	3.67388500	-1.52342800	0.00052800
H	5.99576700	-0.73098200	0.00060300
H	5.18138000	1.79158500	-0.00043500
N	1.14686700	-0.60616000	-0.00008200
N	0.33461100	0.36877600	-0.00001800
C	-1.02679700	0.07681300	-0.00015100
C	-1.91062600	1.17234200	0.00010200
C	-1.55543800	-1.23788700	-0.00056900
C	-3.28942500	0.98292600	-0.00001500
H	-1.49874900	2.18377100	0.00035300
C	-2.92220600	-1.43224500	-0.00067400
H	-0.87063500	-2.08627600	-0.00078200
C	-3.80854800	-0.32627300	-0.00037100
H	-3.94946600	1.84946600	0.00010500
H	-3.35124800	-2.43625800	-0.00101500
O	-5.11598700	-0.62459400	-0.00064900
C	-6.08377100	0.41806000	0.00126500
H	-7.06416100	-0.07360800	0.00247100
H	-5.99129400	1.04813100	0.90094000
H	-5.99396300	1.04891000	-0.89815200
H	2.47575800	2.79167400	-0.83019800
H	1.30025300	1.87065800	-0.00014600
H	2.47576400	2.79167400	0.82949600
N	2.31154300	2.20487900	-0.00035100

Azothio-c

C	4.75270100	-0.55577600	0.00765200
C	4.39241100	0.77846800	-0.00265000
C	2.99249000	0.92640800	-0.00617800
C	2.28999800	-0.27792000	0.00160400
S	3.40405800	-1.61616900	0.01298700
H	5.76285800	-0.96320700	0.01223100
H	5.10429400	1.60418300	-0.00758100
N	0.94182300	-0.54802000	0.00183900
N	0.18203500	0.46240400	-0.00112300
C	-1.20173400	0.21196600	-0.00214400

C	-2.03756100	1.34254100	0.01898400
C	-1.76035800	-1.08255300	-0.02342600
C	-3.42045400	1.19076500	0.02145300
H	-1.58888800	2.33720100	0.03478400
C	-3.13890900	-1.23736100	-0.02238900
H	-1.10063600	-1.94975200	-0.04123100
C	-3.97836000	-0.10140300	0.00068300
H	-4.07040800	2.06664500	0.03928000
H	-3.57904300	-2.23574900	-0.03953000
C	-5.40419500	-0.26646700	0.00261600
N	-6.55800800	-0.40091600	0.00438500
H	1.24542200	1.94033000	-0.01457300
H	2.48324900	2.77954400	0.80056100
H	2.47875200	2.75677000	-0.85920600
N	2.27462100	2.19378000	-0.02077900

Z isomers, B3LYP/def2svp

Azothio-a

C	2.18227500	2.02580900	-0.24555900
C	3.20562000	1.11255200	-0.10580200
C	2.68443700	-0.18762500	0.03443100
C	1.29663900	-0.28368500	0.01289900
S	0.61230000	1.32112400	-0.19159500
H	2.27902100	3.10388800	-0.37391100
H	4.26467500	1.36806000	-0.10856900
N	0.75045400	-1.55915100	0.05532400
N	-0.45268200	-1.88823700	0.01203700
C	-1.50966600	-0.93054500	0.03337900
C	-2.33232400	-0.80888600	-1.09825300
C	-1.81823700	-0.23344300	1.21424300
C	-3.43165900	0.05103200	-1.06041100
H	-2.09745000	-1.38280600	-1.99702500
C	-2.93577700	0.60424100	1.24514000
H	-1.19674600	-0.36387400	2.10278700
C	-3.73775800	0.75717900	0.10904000
H	-4.06000000	0.16043100	-1.94758100
H	-3.17998000	1.13988600	2.16565800
H	-4.60748700	1.41751200	0.13784400
H	4.00695600	-1.45098200	1.03985900
H	2.76861500	-2.19190500	0.17830800
H	4.11200200	-1.56051300	-0.61468700
N	3.45827000	-1.41225100	0.16809600

Azothio-b

C	-2.68171600	2.15191100	-0.56386500
C	-3.78270900	1.42140100	-0.17681700
C	-3.40317300	0.10864900	0.16874400
C	-2.04447200	-0.17635800	0.06800300
S	-1.20365900	1.26589900	-0.48599900
H	-2.66404900	3.19124200	-0.89134100
H	-4.80150400	1.80708300	-0.15092600
N	-1.67364800	-1.50403100	0.23932400

N	-0.54271600	-2.03255200	0.13849400
C	0.66163100	-1.30019900	0.03837100
C	1.03318800	-0.30996500	0.96432900
C	1.60025500	-1.72129800	-0.92788400
C	2.29400900	0.28457600	0.90291300
H	0.35142600	-0.02630100	1.76843400
C	2.83736700	-1.10400200	-1.01971600
H	1.32909800	-2.52687300	-1.61336200
C	3.20026200	-0.09172300	-0.10545000
H	2.56199600	1.03209000	1.64901600
H	3.55931300	-1.39949700	-1.78351400
O	4.42993000	0.43591000	-0.26154800
C	4.87512900	1.45740000	0.61880800
H	5.88566300	1.72956700	0.28943400
H	4.22502900	2.34669400	0.56574600
H	4.91849500	1.10107300	1.66164100
H	-3.70187400	-1.82445700	0.61410400
H	-4.73425200	-0.82081500	1.48416600
H	-5.05649800	-1.13240600	-0.11393100
N	-4.29683100	-0.96933300	0.56279200

Azothio-c

C	2.33825900	2.24650700	-0.02452200
C	3.51597300	1.52830200	-0.00552400
C	3.24788700	0.14758200	0.00725000
C	1.90002000	-0.19828300	-0.00064200
S	0.92696100	1.26489300	-0.02498000
H	2.23317200	3.33129500	-0.03835200
H	4.50900300	1.97624800	-0.00197500
N	1.58080400	-1.54518700	0.00185800
N	0.44538300	-2.06109800	-0.00303300
C	-0.75154300	-1.28826000	-0.00052000
C	-1.39116900	-0.99867400	-1.21762700
C	-1.35618700	-0.94699700	1.22126300
C	-2.61687200	-0.33951900	-1.21325900
H	-0.92435200	-1.29188400	-2.15948500
C	-2.58276300	-0.28940900	1.22401300
H	-0.86339200	-1.20040000	2.16146600
C	-3.21945300	0.02297800	0.00714200
H	-3.11216500	-0.10538700	-2.15702700
H	-3.05180000	-0.01679300	2.17085600
C	-4.48356000	0.70276400	0.01088800
N	-5.50594800	1.25450100	0.01386400
H	4.83107900	-0.89273800	0.87342700
H	3.72652600	-1.81576600	0.01037100
H	4.87631900	-0.88445200	-0.78626100
N	4.24271600	-0.91398100	0.02672200

Z-twist isomers, B3LYP/def2svp

Azothio-a

C	3.23386500	0.92391700	0.54770600
C	2.34963200	1.61316300	-0.24262800

C	1.29066800	0.78192800	-0.69358800
C	1.36323300	-0.52944300	-0.24402200
S	2.80938300	-0.73973800	0.70744300
H	4.11547100	1.31126200	1.05607800
H	2.44734700	2.67044400	-0.49264200
N	0.66102100	-1.69570600	-0.59503100
N	-0.58327600	-1.79528800	-0.63752100
C	-1.48020400	-0.81960700	-0.11790400
C	-2.64233800	-0.55613500	-0.86947400
C	-1.32742700	-0.25703100	1.16655400
C	-3.61159600	0.31516100	-0.36764100
H	-2.76520800	-1.03910400	-1.84113400
C	-2.32239800	0.58098600	1.66990200
H	-0.45616300	-0.50163200	1.77568100
C	-3.45623300	0.88034300	0.90309900
H	-4.50031500	0.53551700	-0.96296000
H	-2.21269200	1.00223900	2.67171500
H	-4.22551600	1.54411800	1.30373900
H	0.35453600	0.90736000	-2.55907700
H	-0.69971800	1.02097900	-1.28252200
H	0.28385100	2.30029500	-1.67515100
N	0.25440700	1.27451400	-1.59957000
Azothio-b			
C	-3.59775200	-1.61262800	-0.00210000
C	-2.78177700	-1.49086600	1.09249500
C	-1.95950300	-0.33554600	1.00928200
C	-2.13874300	0.41552000	-0.14405100
S	-3.39709100	-0.30660100	-1.11425200
H	-4.30575100	-2.41023200	-0.22120600
H	-2.76411700	-2.19676800	1.92386700
N	-1.70333800	1.69855600	-0.50604300
N	-0.51784900	2.10397000	-0.45865100
C	0.61822500	1.28406400	-0.27832300
C	0.77480800	-0.00828800	-0.82608800
C	1.73252900	1.90001200	0.34016700
C	1.99075900	-0.67891000	-0.72406100
H	-0.04026300	-0.48059500	-1.37527700
C	2.93079300	1.22070800	0.47915600
H	1.62311000	2.91970100	0.71475500
C	3.07704500	-0.07898700	-0.05339200
H	2.08882500	-1.66386700	-1.17918000
H	3.78645200	1.67999700	0.97728100
O	4.27799700	-0.65375300	0.10789800
C	4.51927600	-1.95798300	-0.40708500
H	5.55539600	-2.20372300	-0.14444400
H	3.84284000	-2.69976900	0.04810600
H	4.40672500	-1.98317900	-1.50330000
H	-0.06610600	0.18577000	1.70910200
H	-1.29945300	0.89727100	2.56550300
H	-0.96698700	-0.70301700	2.78789500

N	-1.02526200	0.03339900	2.07123000
Azothio-c			
C	3.50692300	-1.55123900	-0.19858100
C	2.61839200	-1.66782300	0.84115700
C	1.76827500	-0.53637500	0.93598000
C	2.00682700	0.43555400	-0.02920500
S	3.35144800	-0.05166800	-1.02839500
H	4.24507100	-2.28114600	-0.52780000
H	2.57163300	-2.52720400	1.51125300
N	1.55805500	1.75306000	-0.20364700
N	0.36627700	2.11846300	-0.15705800
C	-0.74569500	1.23483000	-0.14826000
C	-1.80438600	1.53679100	0.73025400
C	-0.88875500	0.20511300	-1.10131400
C	-2.97405600	0.78217800	0.69839800
H	-1.69628300	2.36710600	1.43039200
C	-2.07002600	-0.52685800	-1.15261000
H	-0.09140200	-0.00063500	-1.81638100
C	-3.11456900	-0.25202400	-0.24679300
H	-3.78633900	1.00408900	1.39201300
H	-2.18827900	-1.31568000	-1.89704300
C	-4.32767900	-1.01831500	-0.29682800
N	-5.30871200	-1.63859500	-0.33610800
H	1.09104100	0.28334200	2.73238900
H	-0.13781700	-0.09381700	1.68345600
H	0.65462900	-1.29355900	2.50922000
N	0.79338900	-0.39961400	2.01733100
